# Assessment of Heat Exposure and Health Outcomes in Rural Populations of Western Kenya by Using Wearable Devices: Observational Case Study

**DOI:** 10.2196/54669

**Published:** 2024-07-04

**Authors:** Ina Matzke, Sophie Huhn, Mara Koch, Martina Anna Maggioni, Stephen Munga, Julius Okoth Muma, Collins Ochieng Odhiambo, Daniel Kwaro, David Obor, Till Bärnighausen, Peter Dambach, Sandra Barteit

**Affiliations:** 1 Heidelberg Institute of Global Health Heidelberg University Hospital Heidelberg University Heidelberg Germany; 2 Charité – Universitätsmedizin Berlin Institute of Physiology Center for Space Medicine and Extreme Environment Berlin Germany; 3 Department of Biomedical Sciences for Health Universita degli Studi di Milano Milan Italy; 4 Centre for Global Health Research KISUMU Kenya Medical Research Institute Kisumu Kenya; 5 Department of Global Health and Population Harvard TH Chan School of Public Health Havard University Boston, MA United States; 6 Africa Health Research Institute KwaZulu-Natal Somkhele South Africa

**Keywords:** wearables, wearable, tracker, trackers, climate, Africa, environment, environmental, heat, weather, exposure, temperature, rural, fitness trackers, climate change, health, heat, sub-Saharan Africa, Kenya, outcome, outcomes

## Abstract

**Background:**

Climate change increasingly impacts health, particularly of rural populations in sub-Saharan Africa due to their limited resources for adaptation. Understanding these impacts remains a challenge, as continuous monitoring of vital signs in such populations is limited. Wearable devices (wearables) present a viable approach to studying these impacts on human health in real time.

**Objective:**

The aim of this study was to assess the feasibility and effectiveness of consumer-grade wearables in measuring the health impacts of weather exposure on physiological responses (including activity, heart rate, body shell temperature, and sleep) of rural populations in western Kenya and to identify the health impacts associated with the weather exposures.

**Methods:**

We conducted an observational case study in western Kenya by utilizing wearables over a 3-week period to continuously monitor various health metrics such as step count, sleep patterns, heart rate, and body shell temperature. Additionally, a local weather station provided detailed data on environmental conditions such as rainfall and heat, with measurements taken every 15 minutes.

**Results:**

Our cohort comprised 83 participants (42 women and 41 men), with an average age of 33 years. We observed a positive correlation between step count and maximum wet bulb globe temperature (estimate 0.06, SE 0.02; *P*=.008). Although there was a negative correlation between minimum nighttime temperatures and heat index with sleep duration, these were not statistically significant. No significant correlations were found in other applied models. A cautionary heat index level was recorded on 194 (95.1%) of 204 days. Heavy rainfall (>20 mm/day) occurred on 16 (7.8%) out of 204 days. Despite 10 (21%) out of 47 devices failing, data completeness was high for sleep and step count (mean 82.6%, SD 21.3% and mean 86.1%, SD 18.9%, respectively), but low for heart rate (mean 7%, SD 14%), with adult women showing significantly higher data completeness for heart rate than men (2-sided *t* test: *P*=.003; Mann-Whitney *U* test: *P*=.001). Body shell temperature data achieved 36.2% (SD 24.5%) completeness.

**Conclusions:**

Our study provides a nuanced understanding of the health impacts of weather exposures in rural Kenya. Our study’s application of wearables reveals a significant correlation between physical activity levels and high temperature stress, contrasting with other studies suggesting decreased activity in hotter conditions. This discrepancy invites further investigation into the unique socioenvironmental dynamics at play, particularly in sub-Saharan African contexts. Moreover, the nonsignificant trends observed in sleep disruption due to heat expose the need for localized climate change mitigation strategies, considering the vital role of sleep in health. These findings emphasize the need for context-specific research to inform policy and practice in regions susceptible to the adverse health effects of climate change.

## Introduction

### Climate Change and Health

Anthropogenic climate change has led to a mean global temperature increase of approximately 1 °C from preindustrial levels, with projections indicating a continued rise if substantial reductions in greenhouse gas emissions are not achieved; this warming trend poses profound health risks in low-and middle-income countries (LMICs) due to limited resources for environmental adaptation [1,2]. An emerging body of research indicates that wearable devices (wearables)—compact, noninvasive electronic devices capable of continuously monitoring various health metrics—may offer valuable insights into assessing the health impacts of climate change, especially in LMICs, where data on climate change and health are limited [3-5]. Climate change disproportionately affects regions such as sub-Saharan Africa, where increased temperatures exacerbate vulnerabilities, adversely impacting human health and agricultural productivity [2,6]. Kenya, the focus of our study, is increasingly vulnerable to climate change, with forecasts anticipating higher temperatures and more frequent extreme weather events; yet, there remains a lack of preparedness for necessary adaptation measures [1,7].

### Need for Nuanced Understanding

In LMIC settings, a more nuanced understanding of individual exposure to extreme weather events and the resulting health outcomes is essential for creating tailored interventions and allocating resources efficiently [2]. Wearable devices, given their ability to monitor health metrics continuously and noninvasively, provide valuable insights into the health risks faced by vulnerable communities due to climate change [4,8]. Numerous large-scale studies in high-income settings have explored the use of wearables in health care [9,10], highlighting their potential as early warning systems for outbreaks of flu-like illnesses, among other applications. Although wearables have been utilized in studies within LMICs, notably in India [11], there is a lack in research concerning their use in other LMICs, particularly for assessing the impacts of climate change [3,4].

### Objective of This Study

**Figure 1:**
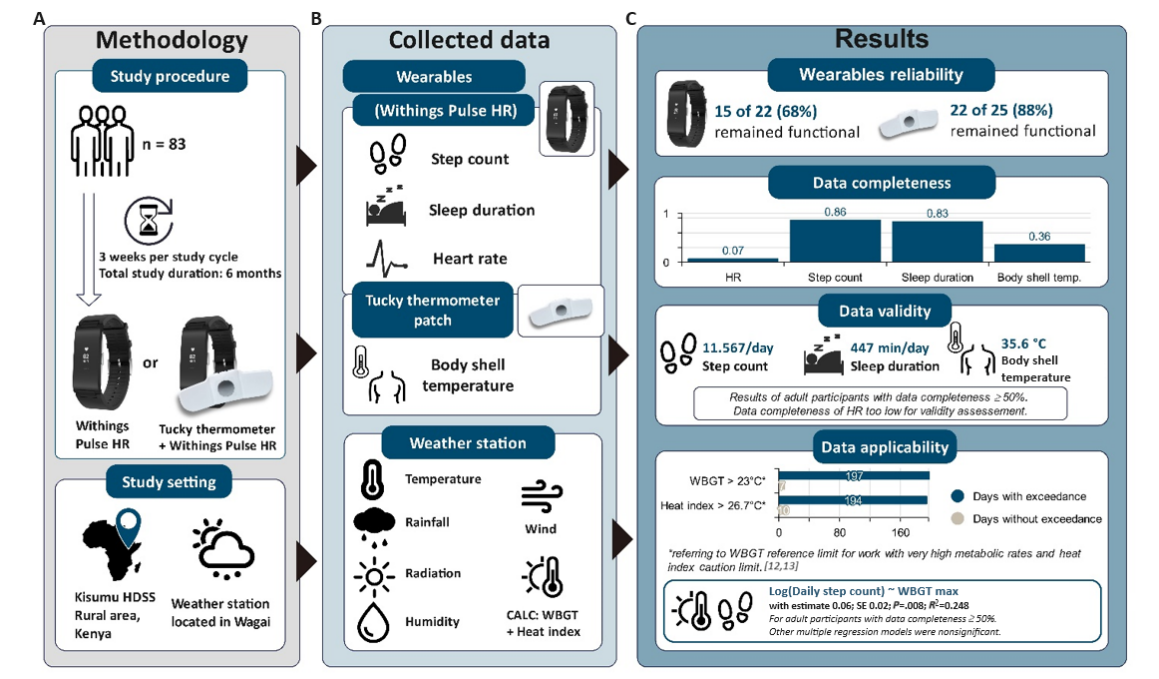
Schematic overview of the observational case study. Reference values are according to Parsons [12] and the National Weather Service [13]. Wearables depiction provided by Withings and e-TakesCare. (A) Methodology involves 83 participants from rural Siaya Health and Demographic Surveillance System, Kenya, equipped with wearables for a 3-week data collection period (total study duration: 9 weeks), coupled with local climate monitoring via a state-of-the-art weather station. (B) Collected data include metrics such as sleep duration, step count, pulse rate, and body temperature, as well as environmental data. (C) Results explore the reliability, completeness, and validity of data, with implications for climate change and health research. HDSS: Health and Demographic Surveillance System; HR: heart rate; WBGT: wet bulb globe temperature.

The primary aim of this study was to assess the feasibility and effectiveness of wearable devices in continuously and objectively monitoring the health impacts of weather exposures on individuals, particularly in a rural setting in Siaya, Kenya (Figure 1). We will integrate these technologies into routine data collection methods of the Health and Demographic Surveillance Systems (HDSS) in Siaya, Kenya, aiming to fill the data void in LMICs by providing measured health metrics that can approximate health impacts, thus offering individual-level, objectively measured health responses to weather exposures.

## Methods

### Ethics Approval

Ethics approval was granted by the Kenya Medical Research Institute (KEMRI/RES/7/3/1) and the ethics committee of the University Hospital Heidelberg, Germany (S-294/2019). This study is reported in accordance with the STROBE (Strengthening the Reporting of Observational Studies in Epidemiology) statement [14].

### Study Design and Participants

This study employs an observational case study methodology in Siaya, a rural county situated in western Kenya, conducted from September 2021 to April 2022. For comprehensive details on the study protocol and a related case study from Burkina Faso, please refer to [15,16]. The Siaya county, about 40 km from Kisumu and 1000 meters above the sea level, hosts the Kenya Medical Research Institute–operated Siaya HDSS, covering an area of 700 km² and serving a population of around 260,000 people [16,17]. The HDSS has over 20 years of retrospective health and demographic data since 1990. Our study’s sample population was stratified by gender, age, and wearable type, with age categories of 6-16 years, 17-45 years, and >45 years. Participants were assigned to either a group with the Withings Pulse Heart Rate (WPHR) wearable or a group using WPHR and the Tucky thermometer patch. Eligibility criteria included age >6 years, living within 5 km of Wagai health center, not planning to move during the study, and providing informed consent. Recruitment methods varied by age, with random selection for those older than 22 years and snowball sampling for those aged 6-22 years.

### Procedures

The study protocol required participants to provide demographic and anthropometric data for wearable calibration, collected during each 3-week study cycle at the Wagai health center. Participants received a compensation of 200 Kenyan shillings (US $1.82) for travel, a smartphone with mobile data for data sync, and a battery pack for charging the wearables. Field personnel conducted weekly visits for data synchronization. Participants wore either the WPHR or both WPHR and a thermometer patch, with WPHR monitoring activity, sleep, and pulse rate, and the patch measuring body shell temperature at night (for detailed information, see Multimedia Appendix 1 [18,19]). The initial inventory consisted of 22 WPHR and 25 thermometer patches. Before reuse, both devices were cleaned and sanitized. This study also used a weather station in Wagai to record various weather parameters. The wet bulb globe temperature (WBGT), indicating heat strain, was calculated using a specific formula incorporating wet bulb temperature, global radiation, relative humidity, and air temperature [20]:


WBGT = (0.7*w) + (0.2*[0.009624*y – 0.00404*z + 1.102*x – 2.2776]) + (0.1*x)

where w represents wet bulb temperature, y represents global radiation, x represents relative humidity, and z represents air temperature.

### Statistical Analysis

Participants were categorized into 4 age groups: school children (6-11 years), adolescents (12-18 years), young adults (19-45 years), and older adults (>45 years). BMI was classified as underweight (<18.5 kg/m^2^), normal weight (18.5-24.9 kg/m^2^), and overweight (>25 kg/m^2^), following World Health Organization guidelines [21]. A descriptive analytical approach was used for demographic details and participant dropouts. BMI for adults was measured at recruitment. Wearable condition and wear were tracked for reliability assessment, and community interviewers’ implementation challenges and infrastructure needs were thematically analyzed.

We analyzed 4 variables to ensure data quality: sleep duration, total step count, heart rate, and body shell temperature. The measured pulse rate was assumed to be equivalent to the participant’s heart rate. We systematically excluded data that showed significant bias or anomalies such as unusually high or low heart rate readings (>expected maximum heart rate or <30 bpm) [22,23] and body shell temperature measurements indicative of protein denaturation or hypothermia-triggered loss of consciousness [24] (detailed criteria for data analysis can be found in Multimedia Appendix 2). Expected maximum heart rate was derived using the equation 208 – 0.7 × age [23]. Based on literature and the WPHR user guide, sleep measurements less than 3 hours (including naps) and those exceeding 13 hours were excluded as nonvalid. Wake times recorded by the wearable were not considered part of sleep duration [25,26]. Sleep episodes exceeding 3 hours were combined if the last episode began before noon on the following day. Differences across gender, study arm, and BMI for adult participants as well as across age groups were analyzed using Welch 2-sided *t* test and Mann-Whitney *U* test, with a 95% CI for error calculation.

To evaluate data completeness, we assessed the proportion of study duration covered by the measurements of the 4 key variables from wearables: sleep duration, step count, heart rate, and body shell temperature. The criteria for data completeness were based on existing literature (detailed in Multimedia Appendix 2 [15,22-28]). For external data validity, we combined individual measurements into a descriptive summary, including data from participants with at least 50% completeness per variable.

We descriptively analyzed the weather profile during the study period and correlations between weather events and adult study participant’s health metrics that had at least 50% data completeness (sleep duration, step count, and body shell temperature). The measurements of WBGT and heat index were categorized using the reference values according to Parsons [12] and the National Weather Service [13]. We employed unadjusted linear regression and multilinear regression analyses [4,8,29-32] considering factors such as maximum daily and minimal nighttime heat and heavy rainfall. The multilinear models further considered gender, age, and BMI as confounders. This study did not account for within-subject trends or the effects of prior day heat stress due to the limitations of the 21-day duration of wearable usage by each participant. Analyses were conducted on R4.1.2 (RStudio version 4.1.2; PBC) with stats3.6.2 for statistics and ggplot2 and car packages for visualization, providing standard error and adjusted *R*^2^ for error quantification and model assessment (see Multimedia Appendix 3 for variable plots and residual plots of the regression models). Numerical values with final digits <5 were rounded down, while numerical values with final digits >4 were rounded up.

## Results

### Demographics of the Participants

**Figure 2:**
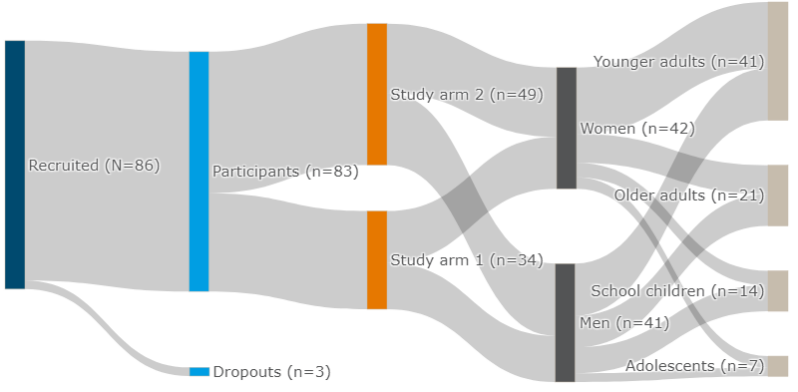
Participant stratification in this study.

We initially enrolled 86 participants in our study; 3 withdrew their consent, resulting in 83 participants for analysis (Figure 2). In the 2 study arms, 34 (41%) participants wore solely the WPHR wearable, while the remaining 49 (59%) wore both the WPHR and the thermometer patch. The mean age of the participants was 33.3 (SD 19) years (range 6-83 years). A further breakdown of this age distribution showed that our study involved 14 school children (17%, age range 6-11 years), 7 adolescents (8%, age range 12-18 years), 41 young adults (49%, age range 19-45 years), and 21 older adults (25%, age >45 years). Women comprised 51% (42/83) of the all the participants. In the adult demographics, which accounted for 75% (62/83) of the participants, the average BMI calculated was 23.8 (SD 4.7) kg/m^2^ (range 16.0-37.4 kg/m^2^).

### Wearables’ Reliability

Of the 22 WPHRs and 25 thermometer patches initially deployed, 7 (32%) WPHRs and 3 (12%) thermometer patches malfunctioned, primarily from physical damage. Technical issues, including data synchronization, were frequent initially but reduced over the study’s duration. Physical damage was the predominant reason for WPHR failures, causing 5 (23%) devices to malfunction due to broken components or overall failure. One WPHR and its charger (5% of the total) were lost. As for thermometer patches, 2 (8%) had damaged charging ports, 1 (4%) was lost, and 2 (8%) had nonretrievable data, despite being intact.

### Data Completeness

Data quality varied across health metrics and participant demographics. Accelerometer metrics such as step count and sleep duration exhibited high completeness, registering 86.1% (SD 18.9%) and 82.6% (SD 21.3%), respectively. Photoplethysmography-based heart rate measurements lagged behind at 7% (SD 14%), while body shell temperature recorded 36.2% (SD 24.5%) completeness. Data completeness was calculated as percentage of study duration covered with measurements in distinct intervals for all participants. Sleep data completeness varied by age: younger adults (19-45 years; n=41) recorded lower data completeness (79%) than older adults (>45 years; n=21; data completeness 90%) (*t* test: *P*=.02; 95% CI –0.20 to –0.02; Mann-Whitney *U* test [MWU] test: *P*=.03; 95% CI –0.14 to 0.00). Further, adult women (n=33) showed less data missingness (87%) than adult men (n=29, 78%) (MWU test: *P*=.03; 95% CI 0.00-0.14). Heart rate data completeness in adult women (12%) was significantly higher than that in men (2%) (*t* test: *P*=.003; 95% CI 0.04-0.17; MWU test: *P*=.001; 95% CI 0.01-0.04). Body shell temperature data completeness for school children (6-11 years; data completeness 47%) was higher than that for adolescents (12-18 years; data completeness 23%) (*t* test: *P*=.03; 95% CI 0.03-0.45; MWU test: *P*=.03; 95% CI 0.05-0.48). After correcting for multiple tests using Holm sequential Bonferroni method [33], only the difference in the completeness of heart rate data between men and women remained statistically significant (*t* test: *P*=.02; MWU: *P*=.004). For detailed data measurements stratified by age, gender, study arm, and BMI, as well as data completeness results corrected for multiple testing, refer to Multimedia Appendix 4 [33].

### Environmental Exposure

**Figure 3:**
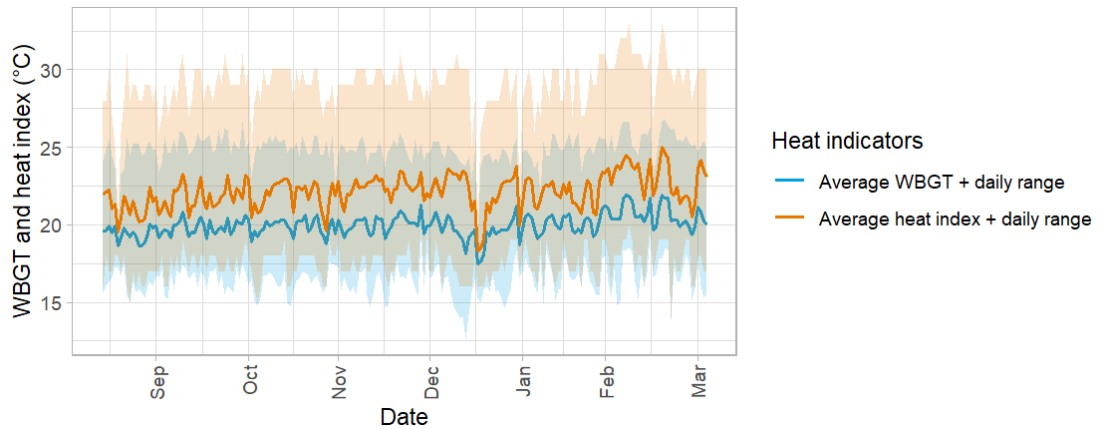
Average daily wet bulb globe temperature in °C (dark blue line) and average daily heat index in °C (orange line) with daily ranges (daily wet bulb globe temperature range in blue ribbon; daily heat index range in orange ribbon). WBGT: wet bulb globe temperature.

During the 204 days study, rainfall was recorded on 97 (47.6%) days. Out of these, heavy rain (≥20 mm per day) was recorded on 16 (7.8%) days. The average daily heat index and WBGT were 22.1 (SD 1.1) °C and 20 (SD 0.7) °C, respectively. The heat index caution limit (26.67 °C or 80 °F) was reached on 194 (95.1%) of 204 days, and the extreme caution limit (32.22 °C or 90 °F) exceeded on only 2 (<1%) days. For the WBGT, reference values for outdoor work with very high metabolic rates were met on 197 (96.6%) days and for work with high metabolic rates on 96 (47.1%) days. Figure 3 illustrates the instances where individual reference values for WBGT, as proposed by Parsons [12], and the heat index, as per the standards of the National Weather Service [13], were exceeded (Table 1).

**Table 1:** Allocation of the respective reference values for wet bulb globe temperature and heat index to the individual risk levels for heat-related diseases depending on the type of work performed and listing of the proportion of days in the observed study period in which these reference values were exceeded. Reference values are according to Parsons [12] and the National Weather Service [13].

Reference			Days with exceedance (n=204), n (%)
**Wet bulb globe temperature reference [12]**			
	Limit for work with a very high metabolic rate (metabolic rate>260 W/m^2^)	197 (96.6)	
	Limit for work with a high metabolic rate (200 W/m^2^<metabolic rate<260 W/m^2^)	96 (47.1)	
	Limit for work with a medium metabolic rate (130 W/m^2^<metabolic rate<200 W/m^2^)	0 (0)	
**Heat index reference [13]**			
	Caution limit (26.67 °C/80 °F)	194 (95.1)	
	Extreme caution limit (32.22 °C/90 °F)	2 (0.1)	
	Danger limit (39.44 °C/103 °F)	0 (0)	

### Data Applicability: Heat Exposure and Health Outcomes

**Figure 4:**
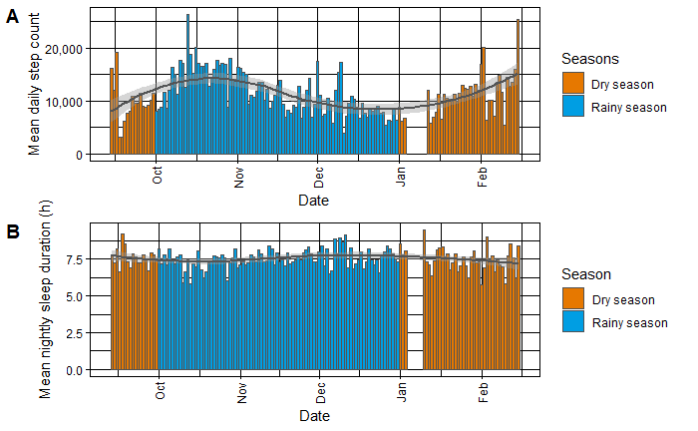
(A) Mean daily step count of adult participants per day and season, wherein dry season is highlighted in orange and rainy season is highlighted in blue. (B) Mean daily sleep duration per adult participant per day and season. Trend lines are added using locally weighted scatterplot smoothing; seasons are classified according to Odhiambo et al [17].

The unadjusted regression analysis for daily step count indicated a significant positive correlation with maximal daily WBGT and heat index (WBGT: estimate 974.4, SE 242.3; *P*<.001; heat index: estimate 317.6, SE 152.4; *P*=.04) with a low *R*^2^ value (WBGT: *R*^2^=0.014; heat index: *R*^2^=0.003). On days without heavy rainfall, a similar positive yet nonsignificant correlation was observed for daily step count (estimate 1466.2, SE 852.9; *P*=.09; *R*^2^=0.002). The analysis for daily maximal temperature did not demonstrate any predictive power for step count (*R*^2^=–0.001; estimate 87.54, SE 145.08; *P*=.55). After a logarithmic transformation of the dependent variable (step count) due to a funnel shape observed in residuals, the confounder-adjusted models revealed a significant positive relationship with the maximum WBGT (estimate 0.06, SE 0.02; *P*=.008; *R*^2^=0.248) (see Figure 4 for details). Other heat indicators showed positive yet nonsignificant associations with daily step count. Age and BMI emerged as significant predictors of daily step count (*P*<.001).

For sleep duration in minutes, the linear regression model revealed a negative, however nonsignificant, correlation with minimal nighttime temperature and heat index at small *P* values, but with low predictive power (temperature: estimate –5.61, SE 3.24; *P*=.08, *R*^2^=0.002; heat index: estimate –4.77, SE 2.91; *P*=.10; *R*^2^=0.002). For minimal nighttime WBGT and sleep duration, simple linear regression had no explanatory power (*R*^2^=–0.001, estimate –0.67, SE 2.98; *P*=.82). Similarly, the multiple linear regression analysis indicated that an increase in minimum nighttime temperatures and nightly minimal heat index corresponded with nonsignificant reductions in sleep duration, that is, by 5.56 minutes per °C increase (*P*=.09; SE 3.234; *R*^2^=0.006) and 4.50 minutes per °C increase (*P*=.12; SE 2.905; *R*^2^=0.006), respectively. We did not detect a significant relationship between nightly minimum WBGT and sleep duration (estimate 0.07 minutes; *P*=.98; SE 2.985; *R*^2^=0.003). Age proved to be a significant determinant of sleep duration across all models (*P*=.03).

Unadjusted linear regression analysis of body shell temperature and minimal nighttime heat had no explanatory power giving their negative adjusted *R*^2^ of –0.003 across all heat indicators (temperature: estimate 0.05, SE 0.07; *P*=.51; WBGT: estimate 0.05, SE 0.07; *P*=.51; heat index: estimate 0.04, SE 0.07; *P*=.53). The multiple linear regression models of nightly body shell temperature did not show significant results either, however at relatively high explanatory power, as indicated by their *R*^2^ values (0.168, 0.170, and 0.170, respectively; temperature: estimate 0.03, SE 0.07; *P*=.63; WBGT: estimate 0.05, SE 0.07; *P*=.42; heat index: estimate 0.05, SE 0.06; *P*=.41, respectively). Gender (*P*<.001) and BMI (*P*=.04) were significant predictors in all 3 models.

The additional analysis incorporating the temporal confounder of weekday or weekend, detailed in Multimedia Appendix 5, did not reveal any significant impact in the models. Regression models were not applied to heart rate measurements, as only the data sets of the 3 participants met the required 50% completeness. A summary of all the multiple regression models, including estimates, standard errors, *P* values, and other considered confounders for each health parameter and the associated extreme weather indicators is provided in Table 2.

**Table 2:** Regression analysis results for health parameters.^a^

Health parameter, variable			Temperature				WBGT^b^				Heat index			Precipitation <20 mm		
			Estimate (SE)		*P* value		Estimate (SE)		*P* value		Estimate (SE)	*P* value		Estimate (SE)		*P* value
**Step count**																
	Intercept	10.47 (0.41)		<.001		9.26 (0.62)		<.001		10.12 (0.45)		<.001	10.79 (0.16)		<.001	
	Gender (men)	0.06 (0.05)		.25		0.05 (0.05)		.28		0.06 (0.05)		.27	0.06 (0.05)		.25	
	Age	–0.02 (0.00)		<.001		–0.02 (0.00)		<.001		–0.02 (0.00)		<.001	–0.02 (0.00)		<.001	
	Maximal daily weather measurement	0.01 (0.01)		.33		0.06 (0.02)		.008		0.03 (0.01)		.08	0.07 (0.08)		.36	
	BMI	–0.03 (0.01)		<.001		–0.03 (0.01)		<.001		–0.03 (0.01)		<.001	–0.03 (0.01)		<.001	
**Sleep duration**																
	Intercept	537.52 (58.98)		<.001		438.98 (52.84)		<.001		520.52 (54.42)		<.001	—^c^		—	
	Gender (men)	1.42 (6.01)		.81		0.93 (6.01)		.88		1.32 (6.01)		.83	—		—	
	Age	–0.39 (0.17)		.03		–0.38 (0.18)		.03		–0.38 (0.17)		.03	—		—	
	Minimal nightly weather measurement	–5.56 (3.23)		.09		0.07 (2.99)		.98		–4.50 (2.91)		.12	—		—	
	BMI	1.02 (0.61)		.09		1.03 (0.61)		.09		1.00 (0.61)		.10	—		—	
**Body shell temperature**																
	Intercept	36.31 (1.34)		<.001		35.98 (1.25)		<.001		36.02 (1.19)		<.001	—		—	
	Gender (men)	–0.72 (0.12)		<.001		–0.72 (0.12)		<.001		–0.72 (0.12)		<.001	—		—	
	Minimum nightly weather measurement	0.03 (0.07)		.63		0.05 (0.07)		.42		0.05 (0.06)		.41	—		—	
	Age	0.00 (0.01)		.41		0.00 (0.01)		.44		0.00 (0.01)		.40	—		—	
	BMI	–0.03 (0.02)		.04		–0.03 (0.02)		.04		–0.04 (0.02)		.04	—		—	

^a^This is a logarithmically transformed model for step count and standard models for sleep duration and body temperature. The table includes estimates, standard errors, and *P* values for each health parameter—sleep duration, step count, and body temperature—along with their associated extreme weather indicators. Health parameters were included of adult participants having at least 50% data completeness for the respective health parameter. Depending on the model, the weather indicators considered were temperature, heat index, wet bulb globe temperature, and precipitation. Additionally, the multiple linear regression models incorporated gender, age, and BMI, as the data validity analysis demonstrated the significance of these confounders.

^b^WBGT: wet bulb globe temperature.

^c^Not available.

## Discussion

### Principal Results

The findings of our study highlight the advantages and challenges associated with the use of wearable devices for the continuous monitoring of vital signs in rural sub-Saharan populations. We found using wearables a pertinent approach for understanding individual impacts of weather exposures. Our research emphasizes the feasibility and effectiveness of integrating wearable technology into health research, in particular, to understand individual exposures and activity patterns such as daily steps and sleep duration. We identified a correlation between weather exposures and various health metrics. Notably, there is a positive relationship between daily step count and the maximum WBGT as well as a potential negative association between nighttime temperatures and sleep duration. These findings contribute to our understanding of the possible health impacts of climate change, with a particular focus on rural regions in western Kenya.

### Comparison With Prior Work

Our study shows environmental exposures such as frequent heavy rainfall and extreme heat, with approximately 20.7% (4044/19,576) of our weather station readings surpassing the heat index caution threshold [13]—a pattern consistent with WBGT findings. We found a strong positive correlation between daily step count and maximum WBGT across both models. Additionally, a positive relationship with the heat index was observed in the unadjusted model. Although many studies indicate a negative link between heat and physical activity [30], the relationship between temperature and activity levels remains inconsistent. This discrepancy necessitates further investigation. For instance, a study [34] conducted in Qatar utilizing wearable pedometers discovered negative correlations between daily step count and both precipitation and temperature. However, that study reported varying associations with WBGT depending on the analytical model employed. Our findings emphasize the need for deeper investigation into this relationship [4]. External factors such as seasonal farming activities illustrated in Figure 4 or specific mitigation practices might significantly influence this correlation and therefore merit additional investigation [17], especially given the scarcity of objectively generated data on activity levels and physical activity profiles in rural sub-Saharan African populations [35].

In our study, we observed a negative trend between minimum nighttime temperature and sleep duration as well as between the minimal nighttime heat index and sleep duration, but these relationships were not statistically significant in either model. However, this trend aligns with previous research that has linked warmer nights to shorter sleep durations, suggesting that climate change could significantly affect sleep health [4,8,32]. For example, a comprehensive study by Minor et al [8] utilizing sleep data of 47,628 participants across 68 countries found a correlation between shorter sleep duration and warmer nights. In line with these findings, all studies in the scoping review of Koch et al [4] reported a negative correlation between sleep duration and heat. These studies underscore the risk of insufficient sleep, in light of the expected impact of global warming due to climate change on local heat exposure [8]. The review of Caddick et al [36] suggests that optimal ambient temperatures for sleep lie between 17 °C and 28 °C, with 40%-60% humidity, although this may vary based on other factors. Insufficient sleep, whether due to short duration or disruptions, can negatively affect human health, potentially compromising the immune system [37] and increasing cardiovascular risk [38].

Regarding body shell temperature, our study did not find significant associations with average nightly body shell temperature and heat. However, previous studies [4,31] have suggested a possible connection. In our analysis, gender and age emerged as critical factors in all 3 models, emphasizing the importance of demographic and physiological factors in body temperature regulation. The inconclusive results in our study might be attributed to the limited sample size and issues with data integrity. Furthermore, natural thermoregulatory processes such as evaporation and the thermoregulatory behaviors of participants should be considered when evaluating body shell temperature [24].

Our study shows improved data completeness compared to previous research in Burkina Faso [15] and the United States [27], especially in thermometer patch data, likely due to enhanced adhesion using medical tape [15]. However, heart rate data showed lower completeness, which may have been affected by technical factors such as the proximity of the sensors to the skin, the impact of motion, and potential device errors. Similar studies using certain wearable devices reported data loss due to wearable malfunctions such as connection issues [39]. Other factors such as blood vessel thickness, skin thickness, obesity, and age might also affect measurement quality and completeness, potentially explaining the gender-specific differences observed in our data, where factors such as thinner skin in women or older populations or thicker blood vessels in men could enhance photoplethysmography signals [40,41]. Other factors that have been mentioned in the scientific literature are the skin pigmentations of the participants; several studies have noted a correlation between the Fitz-Patrick skin scale and heart rate measurements via photoplethysmography, suggesting reduced heart rate measurement accuracy in individuals with higher Fitz-Patrick scale values [40,42], although some studies [43] did not find this correlation. To mitigate this, wearables are starting to implement enhanced photoplethysmography sensors or infrared measurements [42]. Moreover, gender may present contradictory effects on the data collection [40]. The influence of BMI on signal quality remains uncertain [40], with additional variables such as blood vessel dilation during physical activity also playing a role [40]. Participant adherence challenges were also observed, as some felt uncomfortable wearing the device continuously, especially at night.

Our research aligns with previous studies on average daily activity and sleep duration, particularly regarding the influence of age [15,32,34]. Although body shell temperature readings aligned with physiological norms, indicating their usefulness in identifying individual anomalies [24], they were notably low, suggesting potential nighttime evaporation processes [24]. As with prior research, factors such as gender and BMI affected body shell temperature [4,24], necessitating further research to comprehensively grasp temperature impacts.

### Limitations

The internal validity of the wearables used in this study was not evaluated; however, models older than the WPHR used here, some requiring manual sleep initiation, have shown largely satisfactory results. Gruwez et al [44] identified a significant correlation between Withings wearables and a research-grade actigraph for step count during daily activities. However, a review by Fuller et al [45] revealed an underestimation of step count in Withings wearables in most examined studies. Comparisons of Withings wearables to polysomnography revealed no significant differences in sleep duration measurements [39], and various studies have noted consistent correlations with polysomnography-recorded total sleep duration [39,44]. Regulatory constraints restricted the recruitment of younger participants in our study, affecting its generalizability. Furthermore, smartphone usage restrictions in Kenyan boarding schools, especially pertinent to the substantial under-15 years demographic, could pose challenges for future research [17]. In addition, as explained in the methods section, only the data sets of the adult participants were analyzed with regard to weather effects. Our study has other limitations such as uncorrected multiple testing and potential influences of the COVID-19 pandemic on data. In Kenya, the COVID-19 pandemic caused governmental restrictions on public life such as nighttime curfew until October 20, 2021, which affected 22 (26.5%) participants during their study participation—likely influencing the data collected [46]. Research during the 2020 COVID lockdowns reported reduced total sleep duration and increased napping [47], and a South African study observed decreased mobility, particularly on weekends [48]. Future research should consider a wider range of variables, account for the carryover effects of prior day heat strain [49], and address technical challenges such as inaccessible raw data, software issues, high data missingness for heart rate due to factors such as skin type [40], and possible algorithmic biases. Our study does not account for factors such as clothing, air circulation, bedding, and individual differences such as gender, which can influence the relationship between heat and sleep duration [36].

### Conclusions

Our study shows the potential of wearable devices to monitor vital signs and assess the impact of environmental exposures on health in rural sub-Saharan settings, with implications for understanding the nuanced effects of climate change. Despite a robust data set, our findings indicate the need for improved wearable technology to ensure data completeness across diverse demographic groups, acknowledging the impact of factors such as age, gender, and BMI. The positive correlation between physical activity levels and high WBGT offers new insights into behavior during extreme weather conditions, while the nonsignificant trends in sleep duration in relation to temperature call for further investigation. These observations are crucial for public health strategies in climate-vulnerable regions, guiding the integration of wearables in longitudinal health monitoring and climate resilience research. Future studies should expand on the relationship between weather and health outcomes, including a broader demographic and addressing technical challenges identified in wearable data collection. This research contributes to a growing body of knowledge that will inform both technological innovation in health monitoring and the development of interventions to mitigate the health impacts of global climate dynamics.

## Appendix

Multimedia Appendix 1 Detailed technical information on the wearables used.

Multimedia Appendix 2 Additional information on data analysis.

Multimedia Appendix 3 Added variables and residual plots of regression models.

Multimedia Appendix 4 Detailed results on data completeness and data validity.

Multimedia Appendix 5 Additional regression models incorporating weekday or weekend.

Multimedia Appendix 6 ChatGPT conversation.

## Abbreviations

HDSS: Health and Demographic Surveillance System
LMICs: low- and middle-income countries
MWU: Mann-Whitney U test
STROBE: Strengthening the Reporting of Observational Studies in Epidemiology
WBGT: wet bulb globe temperature
WPHR: Withings pulse heart rate

## Data Availability

Deidentified wearable data, weather data, and R scripts will be available for scientific purposes with publication on reasonable request to the corresponding author SB (barteit@uni-heidelberg.de).
