# Supplementary material for: Assessment of Heat Exposure and Health Outcomes in Rural Populations of Western Kenya by Using Wearable Devices: Observational Case Study
Source: JMIR Mhealth Uhealth. 2024 Jul 4;12:e54669. doi: 10.2196/54669 (PMC11258525; doi:10.2196/54669)
Supplement: Multimedia Appendix 1 [file mhealth_v12i1e54669_app1.docx]

**Multimedia Appendix 1: Detailed technical information on wearables used**

1. **Detailed technical information on wearables used:** Wearables depiction provided by Withings and eTakes-Care.

| Wearable | | WPHR | Thermometer patch |
| --- | --- | --- | --- |
| Product name | | Withings pulse HR | Tucky |
| Manufacturer | | Withings | e-TakesCare |
| Hardware specifications | **Appearance** | 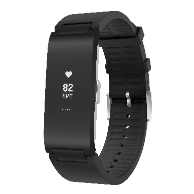 | 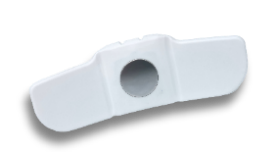 |
|  | **Wear position** | Wrist, bracelet-like | Right axilla, attached with medical tape |
|  | **In-built sensors** | - Accelerometer - Photoplethysmography sensor | - Contact thermistor - (Accelerometer; non-use in study) |
|  | **Further specifications** | - Battery life of up to 21 days - Internal storage capacity for up to 5 days - Waterproof | - Battery life of up to 5 days - Small internal storage - Sweat resistant |
| Software specifications | **Measured health parameters** | Accelerometer:   - Activity (steps, elevation, distance walked, calories burned) - Sleep (length, deepness, onset/offset)   Photoplethysmography sensor   - HR (in beats per minute (bpm)) | Contact thermistor:   - Body shell temperature, rectal equivalent temperature may be calculated by adding 0.7°C to the measured BST |
|  | **Temporal resolution** | Accelerometer:   - Continuously   Photoplethysmography sensor:   - HR every 10 minutes, if an ongoing activity is detected, resolution increases to every second | - Measurement at one-minute intervals |
|  | **Data transfer** | Bluetooth transfer to “health mate” application on the participant’s smartphone, upload to Withings’ servers | Bluetooth transfer to “Tucky” application on the participant’s smartphone, upload to e-TakesCare’s servers |
|  | **Data access** | Online access to Withing’s “health mate” web platform, data downloadable manually or via API | Online access to e-TakesCare’s web platform, data downloadable manually |
| Further information | | [18] | [19] |
