# Supplementary material for: Assessment of Heat Exposure and Health Outcomes in Rural Populations of Western Kenya by Using Wearable Devices: Observational Case Study
Source: JMIR Mhealth Uhealth. 2024 Jul 4;12:e54669. doi: 10.2196/54669 (PMC11258525; doi:10.2196/54669)
Supplement: Multimedia Appendix 2 [file mhealth_v12i1e54669_app2.docx]

**Multimedia Appendix 2: Additional information on data analysis:**

- 1. **Overview of criteria of data excluded deemed significantly biased or aberrant based:**

| Analysed variable | Criteria for valid data |
| --- | --- |
| Nighttime measurements - general | Nighttime measurements (sleep and body shell temperature) were included for each night of study participation.  Sleep and body shell temperature measurements from 12:00 onwards were assigned to the subsequent night. |
| Daytime measurements -general | To minimize bias, 20 days (for step count and HR analysis) of the study cycle were analyzed per participant, excluding the recruitment day and the final study day. |
| Sleep duration | Sleep duration measurements shorter than 3 hours or longer than 13 hours were excluded as non-valid, based on literature and product user guide information [25, 26]. |
| Step count | Step measurements with a value of zero were excluded since the data output cannot distinguish between no measurement and a zero value, according to the manufacturer. |
| Body shell temperature | Body shell temperature measurements incompatible with conscious life, precisely body shell temperature measurements <31.3°C or >41.3°C , were also excluded since indicative of protein denaturation or hypothermia-induced loss of consciousness [24].  Body shell temperature measurements <3h and >13h were excluded for consistency with sleep duration measurements |
| HR | HR measurements <30bpm and higher than the expected maximum HR were excluded as incompatible with conscious life. Expected maximum HR was calculated using the equation 208-0.7*Age [22,23] |

- 1. **Overview of wearables, their sensors, measured variables, time interval criteria, criteria for complete data classification, and their rationale:**

|  | In-built sensor | Health parameter | Maximal interval between valid measurements | Rationale for selection | Criteria for complete data | Rationale for selection |
| --- | --- | --- | --- | --- | --- | --- |
| WPHR | Accelerometer | Sleep duration | None (access to already aggregated data only) | - | Proportion of nights with ≥3h and ≤13h of sleep duration measured | [25] |
|  | Accelerometer | Step count | 60 minutes | [15,27,28] | Proportion of days with ≥10h of measurements | [27,28] |
|  | Photoplethysmo-graphy sensor | HR | 15 minutes | [15] | Proportion of study duration covered | [15] |
| Thermometer patch | Contact thermistor | Body shell temperature | 90 seconds | Analogously to the other non-continuously measuring sensor, the photoplethysmography sensor, the manufacturer-specified measurement interval was multiplied by 1.5 | Proportion of nights with ≥3h and ≤13h of continuous measurements | [25] |
