# Supplementary material for: Assessment of Heat Exposure and Health Outcomes in Rural Populations of Western Kenya by Using Wearable Devices: Observational Case Study
Source: JMIR Mhealth Uhealth. 2024 Jul 4;12:e54669. doi: 10.2196/54669 (PMC11258525; doi:10.2196/54669)

**Multimedia Appendix 3: Added variables and residual plots of regression models**

1. **Added variable and residual plots of multiple linear regression models for impact assessment of extreme weather events on momentary health parameters:**
   1. **Added variable and residual plots – step count (log transformed)**


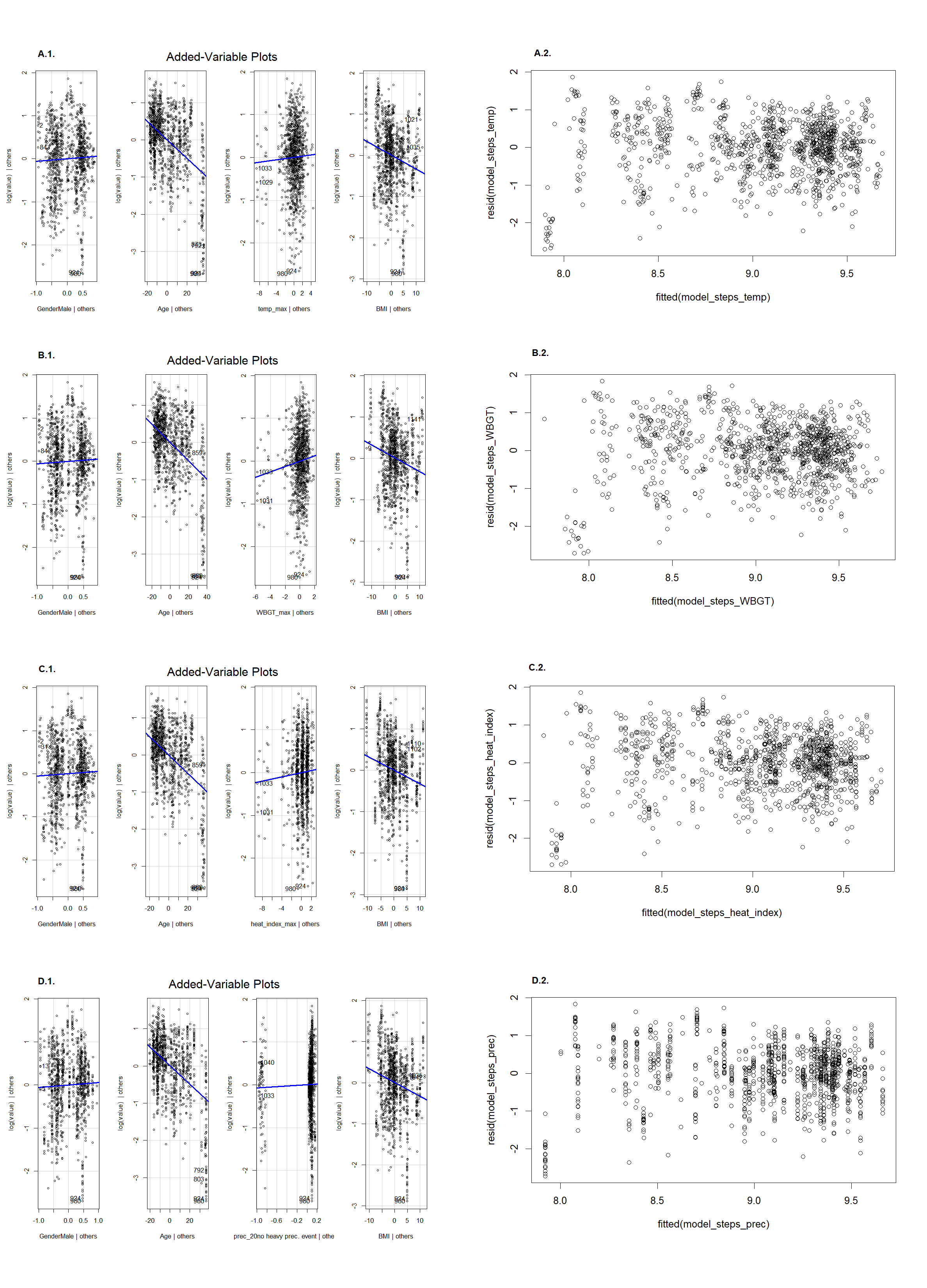


- 1. **Added variable and residual plots – sleep duration**


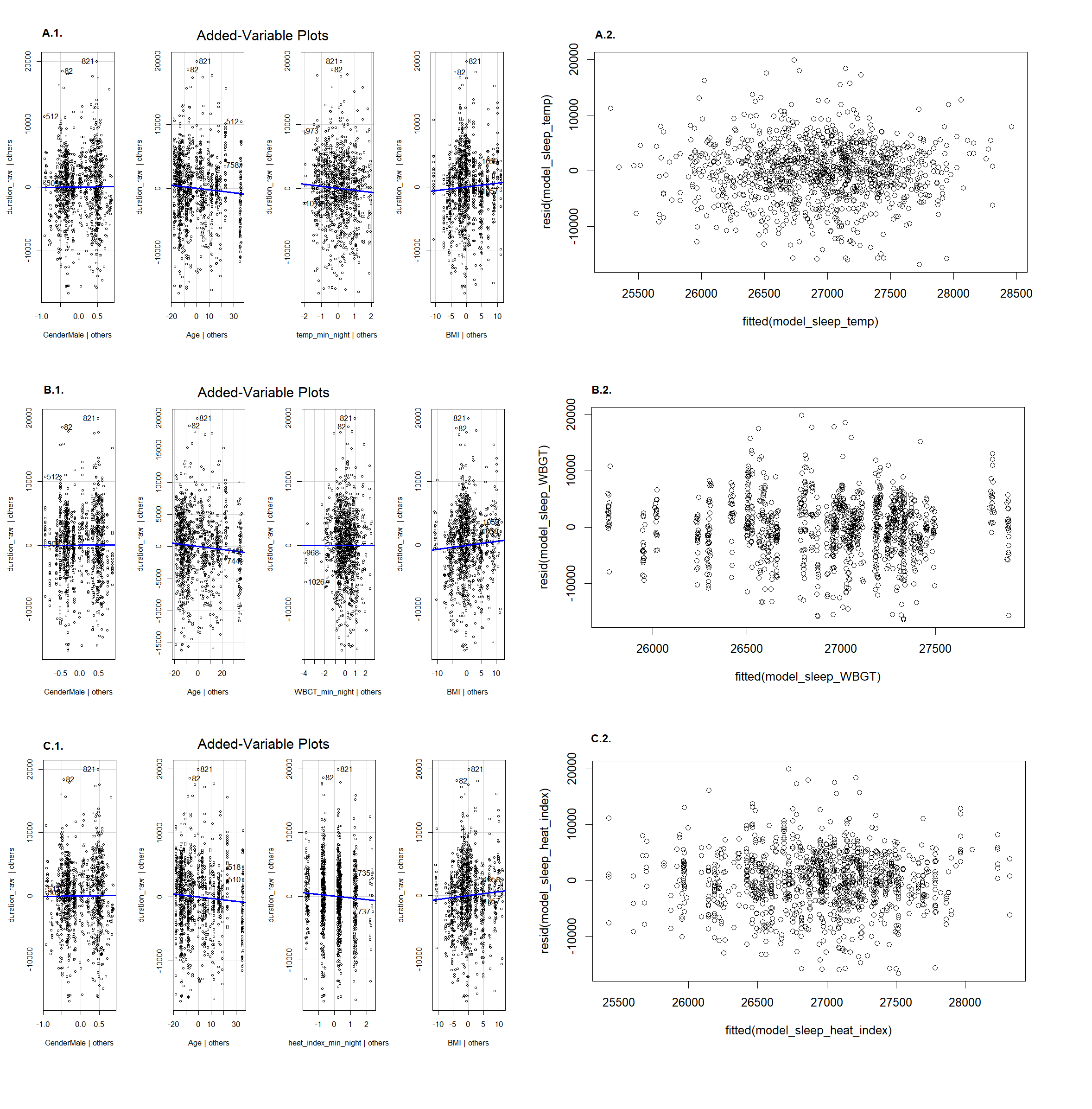


- 1. **Added variable and residual plots – body shell temperature:**


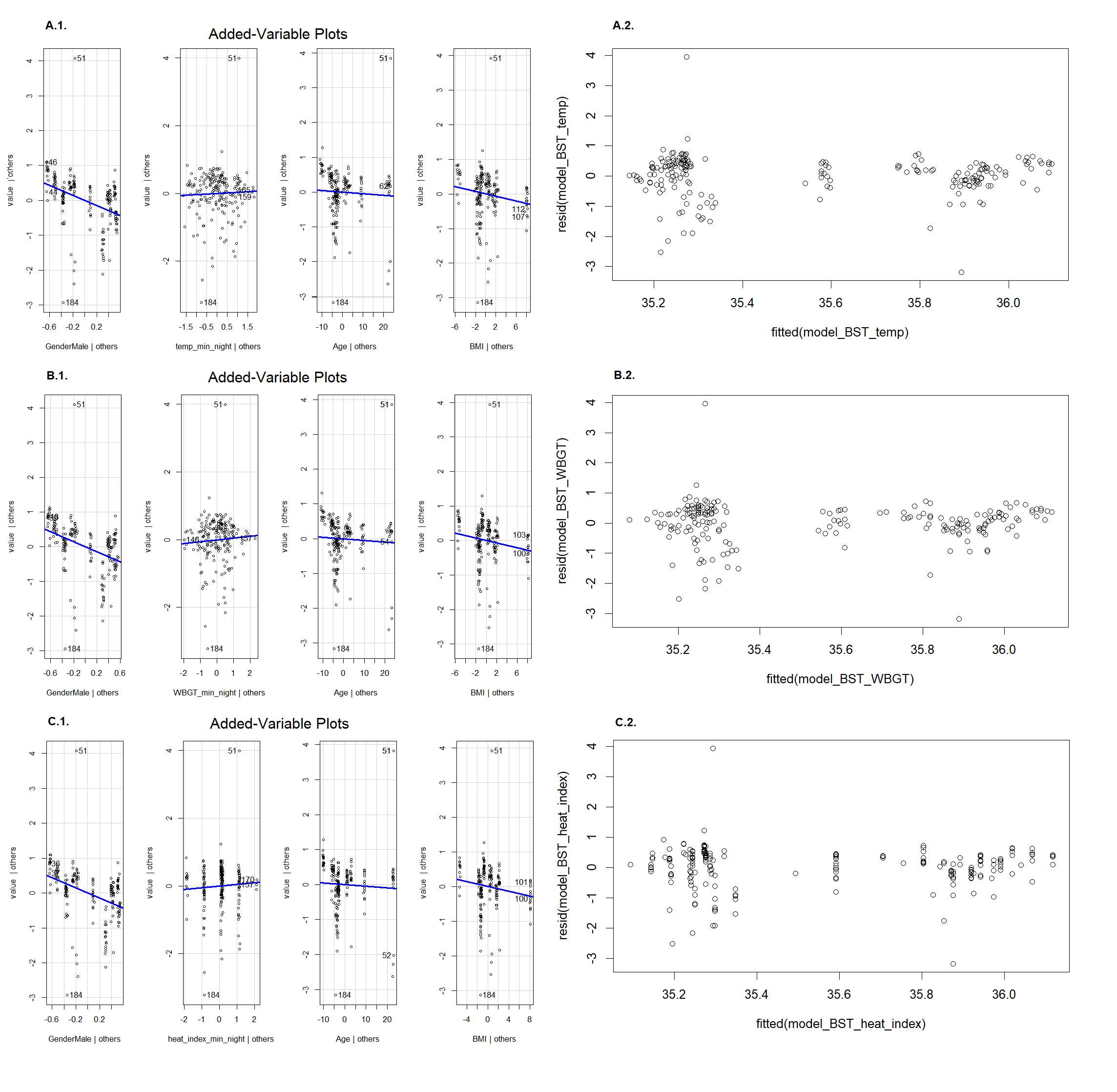

Supplement: Multimedia Appendix 3 [file mhealth_v12i1e54669_app3.docx]
