# Supplementary material for: Assessment of Heat Exposure and Health Outcomes in Rural Populations of Western Kenya by Using Wearable Devices: Observational Case Study
Source: JMIR Mhealth Uhealth. 2024 Jul 4;12:e54669. doi: 10.2196/54669 (PMC11258525; doi:10.2196/54669)
Supplement: Multimedia Appendix 4 [file mhealth_v12i1e54669_app4.docx]

**Multimedia Appendix 4: Detailed results on data completeness and data validity:**

- 1. **Detailed results data completeness:**

|  |  |  |  | Descriptives | | | | Welch-T-Test | | | | Mann-Whitney-U-Test | | |
| --- | --- | --- | --- | --- | --- | --- | --- | --- | --- | --- | --- | --- | --- | --- |
|  |  | |  | **Mean** | **SD** | **Range** | **n** | **t** | **df** | **p** | **95CI** | **W** | **p** | **95CI** |
| Accelerometer | | | |  |  |  |  |  |  |  |  |  |  |  |
|  | **Step count** | | |  |  |  |  |  |  |  |  |  |  |  |
|  |  |  | **Overall** | 0.86 | 0.19 | 0.05;1.00 | 83 |  |  |  |  |  |  |  |
|  |  |  | **Adults** | 0.87 | 0.16 | 0.35;1.00 | 62 | 0.845 | 25.526 | 0.406 | -0.072; 0.173 | 693.5 | 0.649 | -0.050; 0.050 |
|  |  |  | **Children** | 0.82 | 0.26 | 0.05;1.00 | 21 |  |  |  |  |  |  |  |
|  |  | **Children** | **School children** | 0.84 | 0.25 | 0.05;1.00 | 14 | 0.359 | 10.317 | 0.727 | -0.240; 0.334 | 47.5 | 0.909 | -0.100; 0.250 |
|  |  | **Children** | **Adolescents** | 0.79 | 0.29 | 0.20;1.00 | 7 |  |  |  |  |  |  |  |
|  |  | **Adults** | **Younger Adults** | 0.86 | 0.18 | 0.35;1.00 | 41 | -1.498 | 59.904 | 0.139 | -0.125; 0.018 | 415.5 | 0.819 | -0.050; 0.050 |
|  |  | **Adults** | **Older Adults** | 0.91 | 0.10 | 0.60;1.00 | 21 |  |  |  |  |  |  |  |
|  |  | **Adults** | **Women** | 0.87 | 0.17 | 0.35;1.00 | 33 | -0.077 | 59.894 | 0.939 | -0.085; 0.079 | 500.5 | 0.750 | -0.050; 0.050 |
|  |  | **Adults** | **Men** | 0.88 | 0.15 | 0.50;1.00 | 29 |  |  |  |  |  |  |  |
|  |  | **Adults** | **Study arm 1** | 0.88 | 0.14 | 0.50;1.00 | 22 | 0.031 | 52.611 | 0.975 | -0.079; 0.082 | 362 | 0.239 | -0.050; 0.000 |
|  |  | **Adults** | **Study arm 2** | 0.87 | 0.17 | 0.35;1.00 | 40 |  |  |  |  |  |  |  |
|  |  | **Adults** | **Normal weight** | 0.86 | 0.17 | 0.45;1.00 | 40 | -0.898 | 32.935 | 0.376 | -0.135; 0.052 | 310 | 0.592 | -0.050; 0.050 |
|  |  | **Adults** | **Overweight** | 0.90 | 0.15 | 0.35;1.00 | 17 |  |  |  |  |  |  |  |
|  | **Sleep duration** | | |  |  |  |  |  |  |  |  |  |  |  |
|  |  |  | **Overall** | 0.83 | 0.21 | 0.10;1.00 | 83 |  |  |  |  |  |  |  |
|  |  |  | **Adults** | 0.82 | 0.20 | 0.10;1.00 | 62 | -0.083 | 29.026 | 0.934 | -0.129; 0.119 | 564 | 0.356 | -0.095; 0.048 |
|  |  |  | **Children** | 0.83 | 0.25 | 0.10;1.00 | 21 |  |  |  |  |  |  |  |
|  |  | **Children** | **School children** | 0.85 | 0.24 | 0.10;1.00 | 14 | 0.395 | 9.991 | 0.701 | -0.236; 0.339 | 48 | 0.938 | -0.143; 0.238 |
|  |  | **Children** | **Adolescents** | 0.80 | 0.30 | 0.24;1.00 | 7 |  |  |  |  |  |  |  |
|  |  | **Adults** | **Younger Adults** | 0.79 | 0.22 | 0.10;1.00 | 41 | -2.509 | 57.113 | 0.015 | -0.205; -0.023 | 285.5 | 0.029 | -0.143; -0.000 |
|  |  | **Adults** | **Older Adults** | 0.90 | 0.14 | 0.38;1.00 | 21 |  |  |  |  |  |  |  |
|  |  | **Adults** | **Women** | 0.87 | 0.17 | 0.29;1.00 | 33 | 1.839 | 52.378 | 0.072 | -0.008; 0.196 | 631 | 0.029 | 0.000; 0.143 |
|  |  | **Adults** | **Men** | 0.78 | 0.22 | 0.10;1.00 | 29 |  |  |  |  |  |  |  |
|  |  | **Adults** | **Study arm 1** | 0.85 | 0.20 | 0.10;1.00 | 22 | 0.813 | 43.795 | 0.421 | -0.064; 0.151 | 475.5 | 0.597 | -0.048; 0.095 |
|  |  | **Adults** | **Study arm 2** | 0.81 | 0.20 | 0.29;1.00 | 40 |  |  |  |  |  |  |  |
|  |  | **Adults** | **Normal weight** | 0.79 | 0.21 | 0.10;1.00 | 40 | -1.954 | 38.744 | 0.058 | -0.208; 0.004 | 230 | 0.052 | -0.143; 0.000 |
|  |  | **Adults** | **Overweight** | 0.90 | 0.17 | 0.29;1.00 | 17 |  |  |  |  |  |  |  |
| Photoplethysmograph | | | |  |  |  |  |  |  |  |  |  |  |  |
|  | **HR** | | |  |  |  |  |  |  |  |  |  |  |  |
|  |  |  | **Overall** | 0.07 | 0.14 | 0.00;0.75 |  |  |  |  |  |  |  |  |
|  |  |  | **Adults** | 0.07 | 0.14 | 0.00;0.75 | 62 | -0.056 | 35.162 | 0.956 | '-0.073; 0.069 | 665 | 0.883 | -0.010; 0.011 |
|  |  |  | **Children** | 0.07 | 0.14 | 0.00;0.60 | 21 |  |  |  |  |  |  |  |
|  |  | **Children** | **School children** | 0.09 | 0.17 | 0.00;0.60 | 14 | 1.215 | 15.834 | 0.242 | -0.043; 0.157 | 56.5 | 0.576 | -0.029; 0.112 |
|  |  | **Children** | **Adolescents** | 0.03 | 0.04 | 0.00;0.09 | 7 |  |  |  |  |  |  |  |
|  |  | **Adults** | **Younger Adults** | 0.05 | 0.11 | 0.00;0.56 | 41 | -1.168 | 26.318 | 0.253 | -0.145; 0.040 | 374 | 0.400 | -0.022; 0.005 |
|  |  | **Adults** | **Older Adults** | 0.10 | 0.19 | 0.00;0.75 | 21 |  |  |  |  |  |  |  |
|  |  | **Adults** | **Women** | 0.12 | 0.18 | 0.00;0.75 | 33 | 3.169 | 32.877 | 0.003 | 0.036; 0.165 | 720 | 0.001 | 0.006; 0.037 |
|  |  | **Adults** | **Men** | 0.02 | 0.02 | 0.00;0.07 | 29 |  |  |  |  |  |  |  |
|  |  | **Adults** | **Study arm 1** | 0.07 | 0.17 | 0.00;0.75 | 22 | 0.152 | 33.404 | 0.880 | -0.078; 0.090 | 442 | 0.977 | -0.013; 0.010 |
|  |  | **Adults** | **Study arm 2** | 0.07 | 0.12 | 0.00;0.57 | 40 |  |  |  |  |  |  |  |
|  |  | **Adults** | **Normal weight** | 0.05 | 0.10 | 0.00;0.57 | 40 | -1.323 | 19.434 | 0.201 | -0.184; 0.041 | 333 | 0.903 | -0.016; 0.013 |
|  |  | **Adults** | **Overweight** | 0.12 | 0.21 | 0.00;0.75 | 17 |  |  |  |  |  |  |  |
| Contact thermistor | | | |  |  |  |  |  |  |  |  |  |  |  |
|  | **Body shell temperature** | | |  |  |  |  |  |  |  |  |  |  |  |
|  |  |  | **Overall** | 0.36 | 0.25 | 0.00;0.90 | 49 |  |  |  |  |  |  |  |
|  |  |  | **Adults** | 0.36 | 0.26 | 0.00;0.90 | 40 | 0.048 | 18.16 | 0.963 | -0.142; 0.149 | 179,5 | 0.990 | -0.191; 0.190 |
|  |  |  | **Children** | 0.36 | 0.17 | 0.05;0.57 | 9 |  |  |  |  |  |  |  |
|  |  | **Children** | **School children** | 0.47 | 0.09 | 0.33;0.57 | 5 | 2.931 | 4.916 | 0.033 | 0.028; 0.452 | 19 | 0.027 | 0.048; 0.476 |
|  |  | **Children** | **Adolescents** | 0.23 | 0.14 | 0.05;0.38 | 4 |  |  |  |  |  |  |  |
|  |  | **Adults** | **Younger adults** | 0.38 | 0.27 | 0.00;0.90 | 30 | 0.829 | 19.062 | 0.417 | -0.109; 0.252 | 170.5 | 0.520 | -0.095; 0.238 |
|  |  | **Adults** | **Older adults** | 0.31 | 0.22 | 0.00;0.67 | 10 |  |  |  |  |  |  |  |
|  |  | **Adults** | **Women** | 0.35 | 0.27 | 0.00;0.90 | 21 | -0.406 | 37.645 | 0.687 | -0.204; 0.136 | 179.5 | 0.586 | -0.238; 0.143 |
|  |  | **Adults** | **Men** | 0.38 | 0.26 | 0.00;0.86 | 19 |  |  |  |  |  |  |  |
|  |  | **Adults** | **Normalweight** | 0.36 | 0.29 | 0.00;0.90 | 27 | 0.017 | 24.679 | 0.986 | -0.168; 0.171 | 134.5 | 0.986 | -0.238; 0.238 |
|  |  | **Adults** | **Overweight** | 0.36 | 0.19 | 0.00;0.62 | 10 |  |  |  |  |  |  |  |

- 1. **Detailed overview of valid average participant data for daily step count, sleep duration in minutes and body shell temperature in °C:**

|  |  |  | Descriptive statistics | | | | Welch-t-test | | Mann-Whitney-U-test | |
| --- | --- | --- | --- | --- | --- | --- | --- | --- | --- | --- |
|  |  |  | **Mean** | **SD** | **Range** | **n** | **p-value**  **(p)** | **95% Confidence Interval** | **p-value (p)** | **95% Confidence Interval** |
| Step count | **Adults** | | 11566.69 | 6467.70 | 344.11; 35541.14 | 60 | 0.099 | -4811.995; 430.697 | 0.062 | -5645.859; 164.800 |
|  | **Children** | | 13757.33 | 4352.72 | 6129.95; 20207.43 | 19 |  |  |  |  |
|  | **Children** | **School children** | 13995.04 | 3838.47 | 7932.72; 20207.43 | 13 | 0.777 | -5257.819; 6763.286 | 0.861 | -4942.056; 6805.109 |
|  |  | **Adolescents** | 13242.31 | 5690.33 | 6129.95; 19883.20 | 6 |  |  |  |  |
|  | **Adults** | **Younger Adults** | 13114.28 | 6250.95 | 3890.22; 35541.14 | 39 | 0.010 | 1102.569; 7740.819 | 0.002 | 1667.140; 7157.033 |
|  |  | **Older Adults** | 8692.58 | 5984.71 | 344.11; 23530.80 | 21 |  |  |  |  |
|  |  | **Women** | 11490.17 | 7608.45 | 1312.53; 35541.14 | 31 | 0.925 | -3495.448; 3178.853 | 0.455 | -3884.600; 1661.728 |
|  |  | **Men** | 11648.47 | 5109.54 | 344.11; 23530.80 | 29 |  |  |  |  |
|  |  | **Study arm 1** | 10044 | 5255.60 | 1312.53; 20764.72 | 22 | 0.137 | -5600.242; 791.759 | 0.214 | -4888.800; 954.600 |
|  |  | **Study arm 2** | 12448.24 | 6989.28 | 344.11; 35541.14 | 38 |  |  |  |  |
|  |  | **Normal weight** | 13221.04 | 6701.02 | 1312.53; 35541.14 | 39 | <0.001 | 3378.931; 8927.281 | <0.001 | 2690.500; 8573.008 |
|  |  | **Overweight** | 7067.93 | 3477.88 | 344.11; 12806.37 | 16 |  |  |  |  |
| Sleep duration [min] | **Adults** | | 447.01 | 48.23 | 356.58; 561.65 | 58 | <0.001 | -68.743; -27.532 | <0.001 | -70.371; -28.132 |
|  | **Children** | | 495.15 | 34.93 | 426.95; 549.26 | 19 |  |  |  |  |
|  | **Children** | **School children** | 491.67 | 42.01 | 426.95; 549.26 | 13 | 0.377 | -36.917; 14.916 | 0.539 | -52.971; 33.238 |
|  |  | **Adolescents** | 502.67 | 7.50 | 491.76; 513.24 | 6 |  |  |  |  |
|  | **Adults** | **Younger Adults** | 449.14 | 49.96 | 363.21; 548.60 | 38 | 0.638 | -20.149; 32.513 | 0.578 | -20.762; 33.049 |
|  |  | **Older Adults** | 442.96 | 45.72 | 356.58; 561.65 | 20 |  |  |  |  |
|  |  | **Women** | 449.74 | 44.38 | 368.60; 561.65 | 32 | 0.643 | -20.169; 32.359 | 0.803 | -23.167; 33.400 |
|  |  | **Men** | 443.65 | 53.28 | 356.58; 546.65 | 26 |  |  |  |  |
|  |  | **Study arm 1** | 435.80 | 35.23 | 368.60; 493.05 | 21 | 0.139 | -41.027; 5.876 | 0.143 | -44.095; 9.714 |
|  |  | **Study arm 2** | 453.37 | 53.65 | 356.58; 561.65 | 37 |  |  |  |  |
|  |  | **Normal weight** | 447.86 | 48.61 | 363.21; 546.65 | 37 | 0.806 | -34.619; 27.137 | 0.877 | -35.367; 31.750 |
|  |  | **Overweight** | 451.60 | 51.05 | 356.58; 561.65 | 16 |  |  |  |  |
| Body shell temperature [°C] | **Adults** | | 35.645 | 0.555 | 34.322; 36.495 | 12 | too little observations | |  |  |
|  | **Children** | | 35.301 | 0.010 | 35.294; 35.308 | 2 |  |  |  |  |
|  | **Children** | **School children** | 35.301 | 0.010 | 35.294; 35.308 | 2 | NA | |  |  |
|  |  | **Adolescents** | NA | NA | NA | 0 |  |  |  |  |
|  | **Adults** | **Younger Adults** | 35.653 | 0.582 | 34.322; 36.495 | 11 | too little observations | |  |  |
|  |  | **Older Adults** | 35.558 | NA | NA | 1 |  |  |  |  |
|  |  | **Women** | 35.952 | 0.341 | 35.555; 36.495 | 6 | 0.055 | -0.016; 1.246 | 0.037 | 0.059; 1.417 |
|  |  | **Men** | 35.337 | 0.579 | 34.322; 35.952 | 6 |  |  |  |  |
|  |  | **Normal weight** | 35.596 | 0.629 | 34.322; 36.495 | 9 | too little observations | |  |  |
|  |  | **Overweight** | 35.910 | 0.241 | 35.739; 36.080 | 2 |  |  |  |  |

- 1. **Overview of complete HR data sets:**

| Participant characteristics | | | | HR measurements | | |
| --- | --- | --- | --- | --- | --- | --- |
| Gender | **Age group** | **Study arm** | **BMI** | **Mean HR** | **SD** | **Range** |
| Woman | Older adult | 1 | 33.13 | 70.80 | 16.60 | 36; 166 |
| Woman | Younger adult | 2 | 22.82 | 82.55 | 22.27 | 53; 185 |
| Woman | School child | 1 | NA | 91.13 | 15.33 | 61;173 |

- 1. **Data completeness and data validity corrected for multiple testing:** The table displays the p-values corrected for multiple testing for the assessments of data completeness and data validity. Holm's sequential Bonferroni method was applied as the correction procedure [33]. In testing for differences in data completeness among subgroups, after correcting for multiple tests, only the difference in the completeness of heart rate data between men and women remained statistically significant (T-test: p=0.020; MWU: p=0.004). Except for the differences found using the Welch-T-test in the daily step count between younger and older adults, the non-multiple testing corrected disparities in data validity endured correction for multiple testing at a significance level of alpha = 0.05.

Results corrected for multiple testing:

| Data completeness | Step count | | | | Sleep duration | | | | Body shell temperature | | | | HR | | | |
| --- | --- | --- | --- | --- | --- | --- | --- | --- | --- | --- | --- | --- | --- | --- | --- | --- |
|  | **Welch-t-test** | | **MWU-Test** | | **Welch-t-test** | | **MWU-Test** | | **Welch-t-test** | | **MWU-Test** | | **Welch-t-test** | | **MWU-Test** | |
|  | **p** | **p adj.** | **p** | **p adj.** | **p** | **p adj.** | **p** | **p adj.** | **p** | **p adj.** | **p** | **p adj.** | **p** | **p adj.** | **p** | **p adj.** |
| Adults/ Children | 0·406 | 1·00 | 0·649 | 1·00 | 0·934 | 1·00 | 0·356 | 1·00 | 0·963 | 1·00 | 0·99 | 1·00 | 0·956 | 1·00 | 0·883 | 1·00 |
| School children/ Adolescents | 0·727 | 1·00 | 0·909 | 1·00 | 0·701 | 1·00 | 0·938 | 1·00 | 0·033 | 0·166 | 0·027 | 0·137 | 0·242 | 1·00 | 0·576 | 1·00 |
| Younger adults/ Older adults | 0·139 | 0·836 | 0·819 | 1·00 | 0·015 | 0·090 | 0·029 | 0·174 | 0·417 | 1·00 | 0·52 | 1·00 | 0·253 | 1·00 | 0·401 | 1·00 |
| Women/ Men | 0·939 | 1·00 | 0·750 | 1·00 | 0·072 | 0·290 | 0·029 | 0·174 | 0·687 | 1·00 | 0·586 | 1·00 | 0·003 | 0·020 | 0·001 | 0·004 |
| Study arm 1/ Study arm 2 | 0·975 | 1·00 | 0·239 | 1·00 | 0·421 | 1·00 | 0·597 | 1·00 | NA | | | | 0·880 | 1·00 | 0·977 | 1·00 |
| Normal weight/ Overweight | 0·376 | 1·00 | 0·592 | 1·00 | 0·058 | 0·290 | 0·052 | 0·209 | 0·986 | 1·00 | 0·986 | 1·00 | 0·201 | 1·00 | 0·903 | 1·00 |
|  |  | | | | | | | | | | | |  |  |  |  |
| Data validity | **Step count** | | | | **Sleep duration** | | | | **Body shell temperature** | | | |  |  |  |  |
|  | **Welch-t-test** | | **MWU-Test** | | **Welch-t-test** | | **MWU-Test** | | **Welch-t-test** | | **MWU-Test** | |  |  |  |  |
|  | **p** | **p adj.** | **p** | **p adj.** | **p** | **p adj.** | **p** | **p adj.** | **p** | **p adj.** | **p** | **p adj.** |  |  |  |  |
| Adults/ Children | 0·099 | 0·397 | 0·062 | 0·246 | <0·001 | <0·001 | <0·001 | <0·001 | too little observations | | | |  |  |  |  |
| School children/ Adolescents | 0·777 | 1·00 | 0·861 | 0·910 | 0·377 | 1·00 | 0·539 | 1·00 | *NA* | | | |  |  |  |  |
| Younger adults/ Older adults | 0·010 | 0·051 | 0·002 | 0·012 | 0·638 | 1·00 | 0·578 | 1·00 | too little observations | | | |  |  |  |  |
| Women/ Men | 0·925 | 1·00 | 0·455 | 0·910 | 0·643 | 1·00 | 0·803 | 1·00 | 0·055 | 0·055 | 0·037 | 0·037 |  |  |  |  |
| Study arm 1/ Study arm 2 | 0·137 | 0·412 | 0·214 | 0·642 | 0·139 | 0·694 | 0·143 | 0·715 | *NA* | | | |  |  |  |  |
| Normal weight/ Overweight | <0·001 | <0·001 | <0·001 | 0·003 | 0·806 | 1·00 | 0·877 | 1·00 | too little observations | | | |  |  |  |  |

- 1. **Graphical display of valid data:** (A) Hourly average of valid heart rate measurements of the three participants with data completeness for heart rate higher than 50%. Nightly dipping of heart rate is visible. (B) Cumulative plot of most common times of waking up (light blue) and falling asleep (orange) for adult participants. (C) Average body shell temperature of adult participants with data completeness for body shell temperature higher than 50% per 30 minutes including only measurements between median falling asleep and waking up times; nocturnal dipping of the measured body shell temperature in the second half of the night is visible. (D) Averaged step count per 15 minutes of adult participants with data completeness for steps higher than 50%.

**
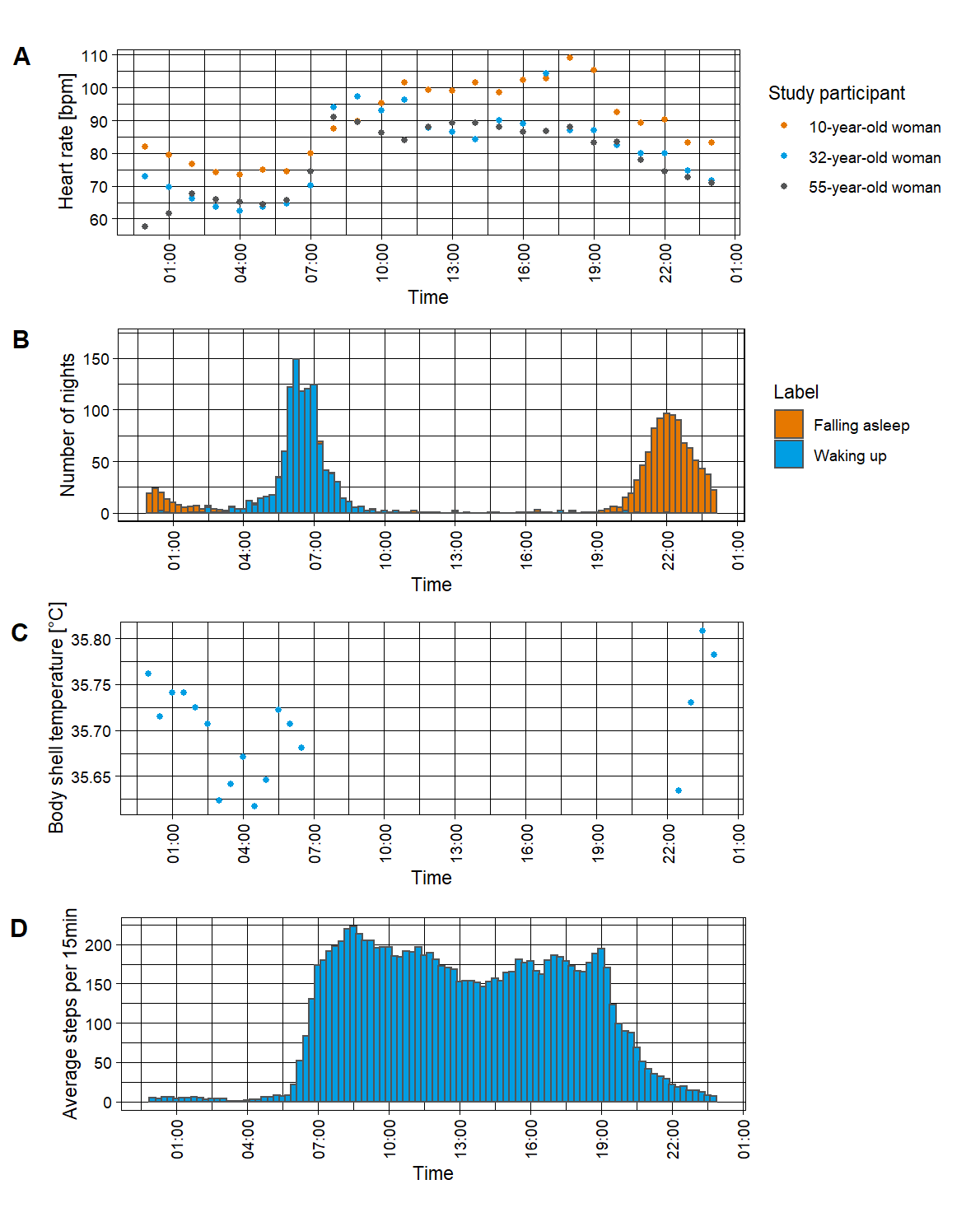
**
