# Supplementary material for: Assessment of Heat Exposure and Health Outcomes in Rural Populations of Western Kenya by Using Wearable Devices: Observational Case Study
Source: JMIR Mhealth Uhealth. 2024 Jul 4;12:e54669. doi: 10.2196/54669 (PMC11258525; doi:10.2196/54669)
Supplement: Multimedia Appendix 5 [file mhealth_v12i1e54669_app5.docx]

**Multimedia Appendix 5: Additional regression models incorporating weekday/weekend**

1. **Additional regression models incorporating weekday/weekend:** Regression analysis results for health parameters include a logarithmically transformed model for step count and standard models for sleep duration and body temperature. The table provides estimates, standard errors, and p-values for each health parameter—sleep duration, step count, and body temperature—along with their associated extreme weather indicators. Depending on the model, weather indicators considered were temperature, heat index, WBGT, and precipitation. Additionally, the multiple linear regression models incorporated gender, age, and BMI, as the data validity analysis demonstrated the significance of these confounders as well as part of the week (weekday/weekend) to assess this possible temporal confounder.

|  | Temperature | | | WBGT | | | Heat index | | | | Precipitation | | | |
| --- | --- | --- | --- | --- | --- | --- | --- | --- | --- | --- | --- | --- | --- | --- |
|  | **Est.** | **SE** | **p** | **Est.** | **SE** | **p** | **Est.** | **SE** | **p** | **Est.** | | **SE** | p |  |
| Step count | | | | | | | | | | | | | | |
| (Intercept) | 10.514 | 0.413 | <0.001 | 9.280 | 0.616 | <0.001 | 10.148 | 0.449 | <0.001 | 10.801 | | 0.161 | <0.001 |  |
| Gender (Men) | 0.058 | 0.050 | 0.250 | 0.054 | 0.050 | 0.279 | 0.055 | 0.050 | 0.274 | 0.0578 | | 0.050 | 0.254 |  |
| Age | -0.025 | 0.002 | <0.001 | -0.025 | 0.002 | <0.001 | -0.025 | 0.002 | <0.001 | -0.025 | | 0.002 | <0.001 |  |
| Maximal daily weather measurement | 0.013 | 0.014 | 0.355 | 0.063 | 0.024 | 0.008 | 0.025 | 0.015 | 0.089 | 0.083 | | 0.082 | 0.316 |  |
| BMI | -0.033 | 0.005 | <0.001 | -0.032 | 0.005 | <0.001 | -0.033 | 0.005 | <0.001 | -0.033 | | 0.005 | <0.001 |  |
| Part of the week (Weekend) | -0.051 | 0.050 | 0.307 | -0.053 | 0.050 | 0.284 | -0.051 | 0.050 | 0.304 | -0.058 | | 0.050 | 0.248 |  |
| Sleep duration | | | | | | | | | | | | | | |
| (Intercept) | 491.050 | 47.885 | <0.001 | 484.873 | 75.692 | <0.001 | 493.114 | 53.579 | <0.001 |  | |  |  |  |
| Gender (Men) | 1.651 | 6.034 | 0.784 | 1.163 | 6.017 | 0.847 | 1.500 | 6.027 | 0.804 |  | |  |  |  |
| Age | -0.391 | 0.175 | 0.026 | -0.392 | 0.175 | 0.026 | -0.392 | 0.175 | 0.025 |  | |  |  |  |
| Minimal nightly weather measurement | -1.930 | 1.632 | 0.237 | -1.847 | 2.907 | 0.525 | -1.911 | 1.765 | 0.279 |  | |  |  |  |
| BMI | 1.079 | 0.614 | 0.079 | 0.983 | 0.615 | 0.110 | 1.022 | 0.612 | 0.095 |  | |  |  |  |
| Part of the week (Weekend) | 8.358 | 6.030 | 0.166 | 8.891 | 6.016 | 0.140 | 8.610 | 6.019 | 0.153 |  | |  |  |  |
| Body shell temperature | | | | | | | | | | | | | | |
| (Intercept) | 36.598 | 1.276 | <0.001 | 35.423 | 1.886 | <0.001 | 35.716 | 1.422 | <0.001 |  | |  |  |  |
| Gender (Men) | -0.718 | 0.123 | <0.001 | -0.728 | 0.123 | <0.001 | -0.725 | 0.123 | <0.001 |  | |  |  |  |
| Age | -0.005 | 0.006 | 0.383 | -0.005 | 0.006 | 0.382 | -0.006 | 0.006 | 0.350 |  | |  |  |  |
| Minimal nightly weather measurement | 0.011 | 0.043 | 0.803 | 0.059 | 0.073 | 0.419 | 0.042 | 0.047 | 0.375 |  | |  |  |  |
| BMI | -0.036 | 0.017 | 0.039 | -0.035 | 0.017 | 0.040 | -0.036 | 0.017 | 0.034 |  | |  |  |  |
| Part of the week (Weekend) | 0.029 | 0.110 | 0.794 | 0.021 | 0.110 | 0.853 | 0.022 | 0.110 | 0.842 |  | |  |  |  |
