# Supplementary material for: Assessment of Heat Exposure and Health Outcomes in Rural Populations of Western Kenya by Using Wearable Devices: Observational Case Study
Source: JMIR Mhealth Uhealth. 2024 Jul 4;12:e54669. doi: 10.2196/54669 (PMC11258525; doi:10.2196/54669)
Supplement: Multimedia Appendix 6 [file mhealth_v12i1e54669_app6.docx]

Disclaimer: The entire ChatGPT chat relating to the period is shown below. Not all aspects have been included in the development of the publication. Also, large parts are in German. ChatGPT was primarily used here as a formulation aid. However, the complete but therefore very detailed and redundant list is intended to provide the greatest possible transparency.

| Authors | What are the temperature recommendations for sleep across the world? |
| --- | --- |
| ChatGPT | Sleep preferences can vary among individuals, and ideal sleeping temperatures can be subjective. However, there are general recommendations based on studies and expert opinions. Keep in mind that personal preferences, clothing, bedding, and local climate can influence what temperature is comfortable for sleep.   1. National Sleep Foundation (NSF) Recommendations:    - The NSF suggests a bedroom temperature between 60-67 degrees Fahrenheit (15-19 degrees Celsius) for optimal sleep. 2. American Academy of Sleep Medicine (AASM) Recommendations:    - The AASM recommends a bedroom temperature between 60-67 degrees Fahrenheit (15-19 degrees Celsius) for adults. 3. World Health Organization (WHO) Recommendations:    - The WHO suggests a temperature range of 18-24 degrees Celsius (64-75 degrees Fahrenheit) for a comfortable and healthy indoor environment, which could include sleep environments. 4. Sleep Council (UK) Recommendations:    - The Sleep Council in the UK suggests a bedroom temperature of around 16-18 degrees Celsius (60-65 degrees Fahrenheit). 5. Environmental Protection Agency (EPA) Recommendations:    - The EPA recommends a bedroom temperature of around 65 degrees Fahrenheit (18 degrees Celsius) for energy efficiency and comfort.   It's important to note that individual preferences can vary, and some people may feel more comfortable with slightly warmer or cooler temperatures. Additionally, factors like bedding, clothing, and personal health conditions can influence the ideal sleeping temperature. Experimenting with different temperatures and paying attention to how well you sleep can help you find the temperature that works best for you. |
| Authors | What are temperature recommendations for sleep in Kenya? |
| ChatGPT | While there might not be specific temperature recommendations for sleep in Kenya, you can consider general guidelines provided by sleep experts. People's preferences for sleeping temperature can vary, but a recommended range for a comfortable sleep environment is typically around 60-67 degrees Fahrenheit (15-19 degrees Celsius).  In Kenya, the climate can vary depending on the region and time of year. Nairobi, for example, has a relatively mild climate, with temperatures often ranging from around 50 to 80 degrees Fahrenheit (10 to 27 degrees Celsius) throughout the year. However, individual preferences, bedding, and other factors can influence what temperature is most comfortable for sleep.  It's essential to consider your personal comfort and adjust your sleeping environment accordingly. Experiment with different temperatures to find what works best for you and ensures a restful night's sleep. Using lightweight and breathable bedding can also contribute to a comfortable sleep experience in warmer climates. |
| Authors | What is 60-67 degrees Fahrenheit in Celcius |
| ChatGPT | To convert Fahrenheit to Celsius, you can use the following formula:  Celsius=(Fahrenheit−32)×59Celsius = (Fahrenheit - 32) \times \frac{5}{9}Celsius=(Fahrenheit−32)×95​  Let's apply this formula to the temperature range of 60-67 degrees Fahrenheit:   1. For 60 degrees Fahrenheit: Celsius=(60−32)×59≈15.56Celsius = (60 - 32) \times \frac{5}{9} \approx 15.56Celsius=(60−32)×95​≈15.56 2. For 67 degrees Fahrenheit: Celsius=(67−32)×59≈19.44Celsius = (67 - 32) \times \frac{5}{9} \approx 19.44Celsius=(67−32)×95​≈19.44   So, the temperature range of 60-67 degrees Fahrenheit is approximately 15.56 to 19.44 degrees Celsius. |
| Authors | Can you link me studies on sleep hygiene and sleep recommendations for subsahara afica? |
| ChatGPT | I'm unable to provide direct links as I don't have browsing capabilities, and my training data doesn't include specific URLs. However, I can guide you on how to find relevant information on sleep hygiene and recommendations for Sub-Saharan Africa.   1. PubMed and Research Databases:    - Use academic databases like PubMed, Google Scholar, or other research databases. You can enter search terms such as "sleep hygiene in Sub-Saharan Africa" or "sleep recommendations Sub-Saharan Africa" to find relevant studies and articles. 2. National Health Organizations:    - Check the websites of health organizations in Sub-Saharan African countries or regional health bodies. They may provide guidelines or links to studies related to sleep hygiene and recommendations. 3. Universities and Research Institutions:    - Explore the websites of universities and research institutions in Sub-Saharan Africa. They often publish research findings and may have studies related to sleep health. 4. International Sleep Associations:    - Organizations like the World Sleep Society or the Sleep Research Society may have global or regional information on sleep recommendations, including Sub-Saharan Africa. 5. Contact Local Health Authorities:    - Reach out to health departments or authorities in Sub-Saharan African countries directly. They might provide information or direct you to relevant studies on sleep hygiene.   When searching, it's important to use specific keywords related to your topic of interest. Additionally, you may need to explore a combination of sources to gather comprehensive information on sleep hygiene and recommendations for Sub-Saharan Africa. |
| Authors | Can you name me studies on sleep hygiene and sleep recommendations for subsahara afica? |
| ChatGPT | I don't have direct access to databases or the ability to provide specific study names. However, I can guide you on how to search for studies on sleep hygiene and sleep recommendations for Sub-Saharan Africa:   1. PubMed and Google Scholar:    - Use academic databases like PubMed or Google Scholar. Enter search terms such as "sleep hygiene Sub-Saharan Africa" or "sleep recommendations Sub-Saharan Africa" to find relevant studies. 2. African Journals Online (AJOL):    - AJOL is a platform that provides access to African-published research. You can search for sleep-related studies on AJOL. 3. World Health Organization (WHO) Publications:    - Check the WHO website or publications for information on sleep recommendations in different regions, including Sub-Saharan Africa. 4. Local Health Ministries and Institutions:    - Explore the websites of health ministries and institutions in Sub-Saharan African countries. They may have published guidelines or research on sleep health. 5. University Research Repositories:    - Look into the research repositories of universities in Sub-Saharan Africa. Universities often publish studies conducted by their researchers. 6. International Sleep Associations:    - Organizations like the World Sleep Society or the Sleep Research Society may have global or regional information on sleep recommendations, including for Sub-Saharan Africa.   When searching, use specific keywords related to sleep, such as "sleep hygiene," "sleep patterns," or "sleep recommendations," along with the region name (e.g., Sub-Saharan Africa). Keep in mind that academic databases and institutional websites are valuable resources for accessing scientific literature. |
| Authors | Can you name me currently published studies working on sleep using wearables? |
| ChatGPT | I don't have real-time access to databases or the internet to provide you with the latest published studies. However, I can guide you on where to find such information.  To discover currently published studies on sleep using wearables, you can follow these steps:   1. PubMed:    - Visit the PubMed database (https://pubmed.ncbi.nlm.nih.gov/) and use search terms such as "sleep wearables," "wearable devices sleep monitoring," or similar keywords to find the latest studies. 2. Google Scholar:    - Use Google Scholar (https://scholar.google.com/) and enter relevant search terms related to sleep and wearables. This can help you find recent publications and academic articles. 3. Research Journals:    - Explore well-known sleep and technology-related journals. Journals such as the "Journal of Sleep Research" or "Sleep Medicine" often feature studies on sleep and wearables. 4. Conferences and Proceedings:    - Look for conference proceedings from events like the World Sleep Congress, Sleep Medicine Conferences, or technology-related conferences. Many studies are presented at conferences before being formally published. 5. Wearable Technology Conferences:    - Explore conferences specifically focused on wearable technology. Researchers often present their findings on sleep monitoring using wearables in these forums. 6. University Research Repositories:    - Check the research repositories of universities, especially those with strong programs in sleep research and technology.   Remember to use specific keywords to refine your search and focus on recent publications. Additionally, some articles may be behind paywalls, so access through academic institutions or other authorized means may be necessary. |
| Authors | What are popular authors for wearables research |
| ChatGPT | Several researchers and experts contribute significantly to the field of wearables and sleep research. While popularity can vary depending on the specific subfield or focus of the research, here are some well-known researchers who have made notable contributions to wearables and sleep studies:   1. Rosie L. Chuang:    - Research interests include sleep disorders, wearable technology, and ambulatory monitoring. 2. Matthew P. Buman:    - Works on the intersection of physical activity, sedentary behavior, sleep, and health, often utilizing wearables. 3. Charlene R. Gamaldo:    - Research involves sleep disorders and the application of technology in sleep medicine. 4. Gilles Vandewalle:    - Known for research on sleep, circadian rhythms, and the impact of light exposure, including studies involving wearables. 5. Daniel A. Klerman:    - Expertise in circadian rhythms, sleep, and the impact of light, often incorporating wearable devices in research. 6. Matteo Pastorino:    - Involved in research related to biomedical signal processing and wearable technologies for monitoring health, including sleep. 7. Susan Redline:    - Research focuses on sleep disorders, sleep epidemiology, and the use of wearable technology in sleep studies. 8. Michael W. L. Chee:    - Research includes cognitive neuroscience, sleep, and the use of technology, including wearables, in sleep research. 9. Mark R. Matthews:    - Works on the development and application of wearable technologies for monitoring sleep and physical activity. 10. Christina M. Spengler:     - Involved in research on chronobiology, sleep, and the impact of light exposure, often utilizing wearables in studies.   Keep in mind that the popularity of researchers can change over time, and new contributors may emerge in this rapidly evolving field. To stay updated, you can follow the latest publications, conferences, and academic events related to wearables and sleep research. |
| Authors | What are popular authors for wearables research on sleep? |
| ChatGPT | As of my last knowledge update in January 2022, several researchers have been actively contributing to the field of wearables research, particularly in the context of sleep. It's essential to note that popularity can change over time, and new researchers may emerge. Here are some researchers who have been known for their work in wearables and sleep research:   1. Dr. Matthew P. Buman:    - Research interests include physical activity, sedentary behavior, and sleep, often utilizing wearables. 2. Dr. Rosalind Picard:    - Known for her work in affective computing and wearable technology, including applications related to sleep monitoring. 3. Dr. Emmanuel Mignot:    - A sleep researcher with a focus on sleep disorders and circadian rhythms, occasionally incorporating wearables in studies. 4. Dr. Jeanne F. Duffy:    - Research involves circadian rhythms, sleep, and the impact of light exposure, with some studies incorporating wearable devices. 5. Dr. Roy J.E.M. Raymann:    - Specializes in sleep, wearables, and chronobiology, contributing to the development of wearable technologies for sleep monitoring. 6. Dr. Christian Cajochen:    - Research includes sleep, circadian rhythms, and the effects of light exposure on sleep, with some studies involving wearables. 7. Dr. Daniel Aeschbach:    - Works on sleep and circadian rhythms, occasionally incorporating wearable technology in research. 8. Dr. Célyne H. Bastien:    - Known for research on sleep disorders, sleep assessment, and the application of technology, including wearables. 9. Dr. Raffaele Ferri:    - Research involves sleep medicine and technology, with contributions to the use of wearables in sleep studies. 10. Dr. Rachel S. Edgar:     - Focuses on sleep research and the application of technology, including wearables, in understanding sleep patterns.   To stay updated on the latest research and discover emerging authors, it's advisable to check recent publications, conference proceedings, and academic journals in the field of sleep and wearables. Additionally, following relevant academic conferences and organizations can provide insights into the current trends and contributors in the field. |
| Authors | Did research studies use the Withings Pulse HR? |
| ChatGPT | I don't have specific information on whether research studies have used the Withings Pulse HR, as my knowledge is limited to information available up until January 2022, and I don't have real-time access to databases. However, researchers commonly use a variety of wearable devices, including those from Withings, in studies related to physical activity, sleep, and overall health monitoring.  If you are interested in finding out if the Withings Pulse HR has been used in research studies, I recommend checking academic databases such as PubMed, Google Scholar, or other research repositories. You can use search terms like "Withings Pulse HR research study" or "wearable device research sleep" to identify relevant studies that may have utilized this specific device. Additionally, you can explore Withings' official publications or contact the company directly for information on any collaborations or studies involving their devices.  Keep in mind that the use of specific devices in research may vary, and researchers often choose devices based on their study objectives, the features of the wearable, and its compatibility with the research goals. |
| Authors | What does psg mean in this sentence: The authors should cite some works regarding the validation of the wearables used in this study in the lab/at home settings against PSG/research grade actigraphs.? |
| ChatGPT | In the context of the sentence you provided, "PSG" stands for Polysomnography. Polysomnography is a comprehensive sleep study that involves monitoring various physiological parameters during sleep, such as brain activity, eye movements, muscle activity, heart rate, respiratory effort, air flow, and oxygen saturation. PSG is often considered the gold standard for assessing sleep architecture and identifying sleep disorders.  In the sentence, the authors are being encouraged to cite works related to the validation of the wearables used in the study. The comparison is suggested against PSG or research-grade actigraphs, which are devices designed to monitor and record physical activity and sleep patterns. Using PSG or research-grade actigraphs helps ensure the accuracy and reliability of the data collected by wearables in both laboratory and home settings. |
| Authors | Translate this to english please |
| ChatGPT | Certainly! The sentence translated to English is:  "The authors should reference some studies that validate the wearables used in this study, comparing them in laboratory or at-home settings against PSG (Polysomnography) or research-grade actigraphs." |
| Authors | - Eine Studie von Fuller et al 2020, die sich reliability und validiy von verschiedenen Werables angeschaut hat, fand bei einer mehrzahl der betrachteten Studien eine Unterschätzung der von Withings Wearables gemessenen Schrittzahl. In diesen Studien wurden jedoch ältere als das hier verwendete Modell eingestzt. |
| ChatGPT | Translation to English:  "A study by Fuller et al. 2020, which examined the reliability and validity of various wearables, found an underestimation of the step count measured by Withings wearables in the majority of the studies considered. However, older models than the one used here were employed in these studies." |
| Authors | Kannst du mir den Z wert eines wilcoxon rang testes erklären? |
| ChatGPT | Natürlich! Der Wilcoxon-Rangsummen-Test, auch bekannt als Wilcoxon signed-rank test, ist ein nicht-parametrischer Test, der verwendet wird, um festzustellen, ob es einen signifikanten Unterschied zwischen zwei gepaarten Gruppen gibt. Der Z-Wert ist eine Statistik, die im Kontext dieses Tests verwendet wird.  Hier sind die Schritte, wie der Z-Wert im Wilcoxon-Test berechnet wird:   1. Rangzuweisung:    - Ordne die absoluten Differenzen zwischen den paarweisen Beobachtungen nach ihrer Größe. Dabei werden die kleinsten Differenzen den Rang 1 erhalten, die nächsten den Rang 2 usw. Bei Gleichständen erhält jede Gruppe den Durchschnitt der Ränge, die sie einnehmen würden. 2. Summe der Ränge der positiven Differenzen:    - Addiere die Ränge der positiven Differenzen. Dies gibt dir die Summe der Ränge für die Beobachtungen, bei denen der Wert in der zweiten Gruppe größer ist als in der ersten. 3. Berechnung des Z-Werts:    - Der Z-Wert wird mit der folgenden Formel berechnet: Z=T−n(n+1)4n(n+1)(2n+1)24Z = \frac{T - \frac{n(n+1)}{4}}{\sqrt{\frac{n(n+1)(2n+1)}{24}}}Z=24n(n+1)(2n+1)​ |
| Authors | was bedeutet ein z wert von -3 |
| ChatGPT | In der Statistik gibt der Z-Wert an, wie viele Standardabweichungen ein bestimmter Datenpunkt oder eine Teststatistik von ihrem Mittelwert entfernt ist. Ein Z-Wert von -3 bedeutet, dass der Datenpunkt oder die Teststatistik drei Standardabweichungen unter dem Mittelwert liegt.  Hier sind einige Interpretationen für Z-Werte:   - **Z-Wert < -1.96 oder Z-Wert > 1.96:** In der Regel wird ein Z-Wert größer als 1.96 oder kleiner als -1.96 als signifikant betrachtet (beim üblichen Signifikanzniveau von 0,05). Ein Z-Wert von -3 fällt in diese Kategorie und würde normalerweise darauf hinweisen, dass die Beobachtung oder der Test signifikant von der erwarteten Norm abweicht. - **Z-Wert < -2 oder Z-Wert > 2:** Ein Z-Wert von -3 liegt über diesem Schwellenwert und zeigt eine noch stärkere Abweichung an. - **Z-Wert < -3 oder Z-Wert > 3:** Ein Z-Wert von -3 liegt sogar weiter außerhalb und deutet auf eine sehr starke Abweichung von der erwarteten Norm hin.   In einem konkreten Anwendungsfall könnte ein Z-Wert von -3 darauf hindeuten, dass der beobachtete Wert sehr weit unter dem Durchschnitt liegt und dies statistisch signifikant ist. Es könnte bedeuten, dass die entsprechende Beobachtung oder Teststatistik in irgendeiner Weise ungewöhnlich oder abweichend ist. Die genaue Interpretation hängt jedoch von der spezifischen Situation und dem Kontext des statistischen Tests ab. |
| Authors | Please translate to englisch: - In Eine weitere Studie die Withings geräte zur schlafaufzeichnung mit polyomnografie verglich, zeigten sich keine signifikanten Unterscheide der gemessenen Schlafdauer sowie eine Korrelation der gesamten Schlafdauer des Withingsgerätes mit der Polysomnografie. Jedoch wurden auch in dieser Studie ältere Withings geräte verwendet, bei denen manuell das Einschlafen angegebn wurde. |
| ChatGPT | Translated to English:  "In another study that compared Withings devices for sleep tracking with polysomnography, no significant differences in measured sleep duration were observed, and there was a correlation between the total sleep duration recorded by the Withings device and polysomnography. However, older Withings devices were also used in this study, where the initiation of sleep was manually indicated." |
| Authors | Kannst du die Sätze bitte auf englisch etwas zusammenfassen und ordentlich ausformulieren? - A study by Fuller et al. 2020, which examined the reliability and validity of various wearables, found an underestimation of the step count measured by Withings wearables in the majority of the studies considered. However, older models than the one used here were employed in these studies. (Fuller et al., 2020) - In another study that compared Withings devices for sleep tracking with polysomnography, no significant differences in measured sleep duration were observed, and there was a correlation between the total sleep duration recorded by the Withings device and polysomnography. However, older Withings devices were also used in this study, where the initiation of sleep was manually indicated. (Mantua et al., 2016) |
| ChatGPT | Certainly! Here are the summarized and rephrased versions of the provided sentences:   1. Regarding Wearables and Step Count (Fuller et al., 2020):    - Fuller et al. (2020) found that in a study examining the reliability and validity of various wearables, Withings wearables consistently underestimated step counts in the majority of cases. It's noteworthy that these studies utilized older Withings models compared to the one used in the present study. 2. Comparing Withings Sleep Tracking and Polysomnography (Mantua et al., 2016):    - In another study comparing Withings devices for sleep tracking with polysomnography, no significant differences were observed in the measured sleep duration. The study indicated a correlation between the total sleep duration recorded by the Withings device and polysomnography. However, it's important to note that older Withings devices were used in this study, and the initiation of sleep was manually indicated. |
| Authors | Bitte umformulieren als weniger sperriger satz - In another study that compared Withings devices for sleep tracking with polysomnography, no significant differences in measured sleep duration were observed, and there was a correlation between the total sleep duration recorded by the Withings device and polysomnography. However, older Withings devices were also used in this study, where the initiation of sleep was manually indicated. (Mantua et al., 2016) |
| ChatGPT | In a different study comparing Withings sleep-tracking devices to polysomnography, there were no significant differences in measured sleep duration. The study found a correlation between the total sleep duration recorded by the Withings device and polysomnography. However, it's worth noting that older Withings devices were used in this study, and the initiation of sleep was manually indicated (Mantua et al., 2016). |
| Authors | For example, a study by Gruwez et al. 2017 compared a Withings wearable to a research grade acitgraph foun a a significant correlation of measured step count in the daily use of the participant. |
| ChatGPT | For instance, a study conducted by Gruwez et al. in 2017 compared a Withings wearable to a research-grade actigraph and found a significant correlation in the measured step count during the participant's daily activities. |
| Authors | kannst du im letzen satz participants daily activities durch etwas ersetzen das beschreibt dass die geräte im normalen umfeld getragen wurden? |
| ChatGPT | Natürlich! Hier ist die angepasste Formulierung:  Zum Beispiel verglich eine Studie von Gruwez et al. aus dem Jahr 2017 ein Withings-Wearable mit einem Actigraph von Forschungsqualität und stellte eine signifikante Korrelation im gemessenen Schrittzähler während des alltäglichen Tragens der Geräte im normalen Umfeld der Teilnehmer fest. |
| Authors | und bitte auf englisch |
| ChatGPT | Certainly! Here's the adjusted sentence in English:  For example, a study by Gruwez et al. in 2017 compared a Withings wearable to a research-grade actigraph and found a significant correlation in the measured step count during the participants' everyday use of the devices in their natural settings. |
| Authors | Please check for mistakes and correct them: - A review by Fuller et al. 2020, which examined the reliability and validity of various wearables, found an underestimation of the step count measured by Withings wearables in the majority of the studies considered (Fuller et al., 2020). However, a study by Gruwez et al. in 2017 compared a Withings wearable to a research-grade actigraph and found a significant correlation in the measured step count during the participants' everyday use of the devices in their natural settings. Another study comparing Withings wearables to polysomnography, there were no significant differences in measured sleep duration. Furthermore, consistent correlations between the total sleep duration recorded by Withings wearables and polysomnography was observed in various studies (Gruwez et al., 2017; Mantua et al., 2016). However, older models than the one used here were employed in these studies which also needed manual indication of the initiation of sleep. |
| ChatGPT | Your original text is well-written, but there are a couple of minor adjustments for clarity and correctness. Here's the revised version:  "A review by Fuller et al. in 2020, which examined the reliability and validity of various wearables, found an underestimation of the step count measured by Withings wearables in the majority of the considered studies (Fuller et al., 2020). However, a study by Gruwez et al. in 2017 compared a Withings wearable to a research-grade actigraph and found a significant correlation in the measured step count during participants' everyday use of the devices in their natural settings. In another study comparing Withings wearables to polysomnography, no significant differences in measured sleep duration were observed. Furthermore, consistent correlations between the total sleep duration recorded by Withings wearables and polysomnography were observed in various studies (Gruwez et al., 2017; Mantua et al., 2016). However, older models than the one used here were employed in these studies, which also required manual indication of the initiation of sleep." |
| Authors | was ist eine multiple comparison corrected analysis? |
| ChatGPT | Eine "multiple comparison corrected analysis" ist eine statistische Analyse, die darauf abzielt, das Problem von multiplen Vergleichen zu behandeln, das bei der gleichzeitigen Durchführung mehrerer statistischer Tests entstehen kann. Das Hauptproblem bei multiplen Vergleichen besteht darin, dass die Wahrscheinlichkeit von zufälligen Fehlern (sogenannten Typ-I-Fehlern oder Alpha-Fehlern) steigt, wenn mehrere Tests durchgeführt werden, und dies kann zu falsch positiven Ergebnissen führen.  Um dieses Problem zu lösen, werden verschiedene Methoden zur Korrektur von multiplen Vergleichen eingesetzt. Einige der gebräuchlichsten Ansätze sind:   1. Bonferroni-Korrektur:    - Diese Methode teilt das Signifikanzniveau (Alpha-Niveau) durch die Anzahl der Vergleiche. Das reduziert die Wahrscheinlichkeit von falsch positiven Ergebnissen, aber es kann zu einer erhöhten Wahrscheinlichkeit von falsch negativen Ergebnissen führen. 2. Holm-Korrektur:    - Ähnlich wie die Bonferroni-Korrektur, aber etwas weniger konservativ, da sie die strengsten Korrekturen auf die am wenigsten signifikanten Tests anwendet. 3. FDR-Korrektur (False Discovery Rate):    - Diese Methode steuert die Wahrscheinlichkeit dafür, dass mindestens ein Vergleich ein falsch positives Ergebnis liefert, im Gegensatz zur Kontrolle der Wahrscheinlichkeit von falsch positiven Ergebnissen insgesamt. 4. Tukey-Korrektur:    - Wird oft in Verbindung mit ANOVA (Analysis of Variance) verwendet und stellt sicher, dass nur signifikante Unterschiede als solche identifiziert werden, wenn es mehrere Gruppen gibt.   Die Wahl der Methode hängt von der spezifischen Forschungsfrage, dem experimentellen Design und den Daten ab. Eine korrekte Anpassung für multiple Vergleiche trägt dazu bei, die Gültigkeit von statistischen Ergebnissen zu erhalten und die Wahrscheinlichkeit von Fehlinterpretationen zu reduzieren. |
| Authors | welche korrektur für multiples testen eignet sich am besten für ein explorative studie mit vielen demographischn unterschieden aber nur wenigen teilnehmern? |
| ChatGPT | In einer explorativen Studie mit vielen demografischen Unterschieden und nur wenigen Teilnehmern kann die Wahl der Korrektur für multiple Tests von verschiedenen Faktoren abhängen. Hier sind einige Überlegungen:   1. Bonferroni-Korrektur:    - Diese Methode ist sehr konservativ und könnte bei kleinen Stichprobengrößen zu starken Anpassungen führen, was die Möglichkeit von falsch negativen Ergebnissen erhöhen könnte. Dennoch bietet sie eine klare Kontrolle über den Alpha-Fehler. 2. False Discovery Rate (FDR)-Korrektur:    - Die FDR-Korrektur (zum Beispiel mit der Benjamini-Hochberg-Methode) ist weniger konservativ als die Bonferroni-Korrektur und könnte für explorative Studien geeignet sein. Sie kontrolliert die Anzahl der falsch positiven Entdeckungen insgesamt, anstatt die Rate der falsch positiven Ergebnisse zu minimieren. 3. Holm-Korrektur:    - Die Holm-Korrektur ist etwas weniger streng als die Bonferroni-Methode, aber sie bietet dennoch eine gewisse Kontrolle über die Familiengröße der Tests. 4. Benjamini-Krieger-Yekutieli (BKY)-Korrektur:    - Eine modifizierte Version der Benjamini-Hochberg-Methode, die in bestimmten Fällen etwas konservativer ist.   Bei kleinen Stichprobengrößen ist es wichtig zu beachten, dass selbst mit einer Korrektur keine Garantie für die Vermeidung von Fehlern besteht. Die Auswahl der Methode hängt von der Toleranz für falsch positive Ergebnisse und der spezifischen Forschungsfrage ab. In explorativen Studien ist es oft sinnvoll, Ergebnisse als vorläufig zu betrachten und weitere Forschung zur Bestätigung durchzuführen. |
| Authors | Wie funktioniert die False discovery rate correktur? |
| ChatGPT | Die False Discovery Rate (FDR)-Korrektur ist eine Methode zur Kontrolle der Rate der falsch positiven Entdeckungen bei der Durchführung von mehreren statistischen Tests. Im Gegensatz zu strengeren Methoden wie der Bonferroni-Korrektur, die die Wahrscheinlichkeit eines einzigen falsch positiven Ergebnisses (Typ-I-Fehler) über alle Tests kontrollieren, zielt die FDR-Korrektur darauf ab, die Anzahl der falsch positiven Entdeckungen relativ zur Gesamtanzahl der positiven Ergebnisse zu kontrollieren.  Hier sind die grundlegenden Schritte der FDR-Korrektur:   1. Führe alle gewünschten Tests durch:    - Führe alle geplanten statistischen Tests durch, ohne eine Korrektur vorzunehmen. 2. Ordne die p-Werte der Tests:    - Ordne die p-Werte der Tests in aufsteigender Reihenfolge. 3. Berechne die kritischen Werte:    - Berechne die kritischen Werte oder Schwelle für Signifikanz. Dies kann auf verschiedene Arten erfolgen, aber eine häufige Methode ist die Benjamini-Hochberg-Prozedur. 4. Vergleiche p-Werte mit den kritischen Werten:    - Vergleiche jeden p-Wert mit dem entsprechenden kritischen Wert. Wenn der p-Wert kleiner oder gleich dem kritischen Wert ist, wird der Test als signifikant betrachtet. 5. Berichte die Ergebnisse:    - Berichte die Ergebnisse der signifikanten Tests nach der FDR-Korrektur.   Die Benjamini-Hochberg-Prozedur ist eine spezifische Methode zur FDR-Korrektur. Sie legt einen kritischen Schwellenwert fest, bei dem p-Werte kleiner oder gleich diesem Wert als signifikant betrachtet werden. Dieser Schwellenwert wird anhand der Anzahl der Tests und der Position des p-Werts in der sortierten Liste berechnet.  Die FDR-Korrektur ermöglicht eine höhere Empfindlichkeit für die Entdeckung von wahren positiven Ergebnissen in explorativen Studien, ist jedoch weniger konservativ als andere Korrekturmethoden. Beachte jedoch, dass eine signifikante Entdeckung nach der FDR-Korrektur nicht unbedingt bedeutet, dass diese Entdeckung sicher ist, sondern dass die Wahrscheinlichkeit eines falsch positiven Ergebnisses kontrolliert wurde. |
| Authors | wie funktioniert die Benjamini-Hochberg-Prozedur |
| ChatGPT | Die Benjamini-Hochberg-Prozedur ist eine Methode zur Kontrolle der False Discovery Rate (FDR) bei der Durchführung mehrerer statistischer Tests. Die FDR-Korrektur ist darauf ausgerichtet, die Rate der falsch positiven Entdeckungen zu kontrollieren, insbesondere wenn viele Tests durchgeführt werden.  Hier sind die Schritte der Benjamini-Hochberg-Prozedur:   1. Führe alle Tests durch:    - Führe alle gewünschten statistischen Tests ohne vorherige Korrektur durch und erhalte die p-Werte für jeden Test. 2. Sortiere die p-Werte:    - Ord |
| Authors | was ist der zweite schritt der Benjamini Hochberg prozedur? |
| ChatGPT | Entschuldigung für das Missverständnis in meiner vorherigen Antwort. Lassen Sie mich den zweiten Schritt der Benjamini-Hochberg-Prozedur genauer erläutern:   1. Sortiere die p-Werte:    - Ordne die p-Werte der einzelnen Tests in aufsteigender Reihenfolge an. 2. Berechne die kritischen Werte:    - Berechne die kritischen Werte (auch als kritische Schwellenwerte bezeichnet). Diese werden basierend auf dem gewünschten FDR-Level und der Anzahl der Tests festgelegt. Der FDR-Level gibt an, wie viele falsch positive Entdeckungen prozentual akzeptabel sind. 3. Vergleiche die p-Werte mit den kritischen Werten:    - Beginne mit dem kleinsten p-Wert und vergleiche ihn mit dem kritischen Wert, der auf Basis des FDR-Level und der Anzahl der Tests berechnet wurde. Wenn der p-Wert kleiner oder gleich dem kritischen Wert ist, wird der entsprechende Test als signifikant betrachtet. 4. Beende den Vergleich:    - Fortsetzen Sie diesen Vergleich schrittweise, bis du den ersten nicht signifikanten p-Wert erreichst. Alle Tests mit p-Werten kleiner oder gleich ihrem kritischen Wert werden als signifikant betrachtet.   Die Benjamini-Hochberg-Prozedur erlaubt eine kontrollierte Handhabung der FDR, wodurch die Wahrscheinlichkeit von falsch positiven Entdeckungen begrenzt wird. Es ist wichtig zu beachten, dass die Benjamini-Hochberg-Prozedur weniger konservativ ist als beispielsweise die Bonferroni-Korrektur, was bedeutet, dass sie mehr Macht für die Entdeckung wahrer positiver Ergebnisse in großen Datensätzen bietet. |
| Authors | Please write in english: Tabelle XX zeigt die für multiples Testen korrigierten p-Werte der Testungen der Data completeness und Data validity. Als Korrekturverfahrne wurde Holms sequentielle Bonferonni Methode verwendet |
| ChatGPT | In English:  "Table XX displays the p-values corrected for multiple testing for the assessments of Data Completeness and Data Validity. Holm's sequential Bonferroni method was applied as the correction procedure." |
| Authors | Please write and correct in english: Bis auf die mit dem Welch-Test gefunden unterschiede der täglichen Schrittzahl von Jungen und älteren erwachsenen, haben die in der nicht auf multiples testen korriegierten Unterschiede der Data validity der Korrektur auf multiples testen bei einem Signifikanzniveau von alpha = 0.05 standgehalten |
| ChatGPT | Here's the corrected sentence in English:  "Except for the differences found using the Welch test in the daily step count between young and older adults, the non-multiple testing corrected disparities in Data Validity endured correction for multiple testing at a significance level of alpha = 0.05." |
| Authors | Please translate to english and reformulate if necessary: Bei der Testung auf unterschiede der Data completeness nach Subgruppen ist bei korrektur auf multiples testen nur der Unterschied der Vollständigkeit der Herzfrequenzdaten bei Männern und Frauen signifikant geblieben. |
| ChatGPT | Translated and reformulated in English:  "In testing for differences in Data Completeness among subgroups, after correcting for multiple tests, only the difference in the completeness of heart rate data between men and women remained statistically significant." |
| Authors | Please translate and correct to english: Extreme Herzfrequenz messungen unter <30bpm wurden als nicht-valide exkludiert da sie nicht mit consciousness vereinbar sind, gleiches gilt für gemessene Herzfrequenzen über der individuellen expected maximum HR |
| ChatGPT | Translated and corrected in English:  "Extreme heart rate measurements below <30 bpm were excluded as non-valid since they are not consistent with consciousness. The same applies to measured heart rates exceeding the individual expected maximum HR." |
| Authors | Can you please write me an R code for an histogram with an vertical median line in ggplot2? |
| ChatGPT | Certainly! Below is an example of R code using ggplot2 to create a histogram with a vertical median line:  # Install and load ggplot2 if not already installed  # install.packages("ggplot2")  library(ggplot2)  # Sample data (replace this with your own dataset)  set.seed(123)  data <- rnorm(100)  # Create a histogram with ggplot2  hist_plot <- ggplot(data, aes(x = data)) +  geom_histogram(binwidth = 0.5, fill = "blue", color = "black", alpha = 0.7) +    # Add a vertical line for the median  geom_vline(xintercept = median(data), color = "red", linetype = "dashed", size = 1) +    # Add labels and a title  labs(title = "Histogram with Median Line",  x = "Values",  y = "Frequency")  # Display the plot  print(hist_plot)  In this example, the geom_histogram function is used to create the histogram, and the geom_vline function adds a vertical line at the median value of the dataset. You can customize the colors, linetypes, and other parameters according to your preferences. |
| Authors | can you give me the r code for ggplot 2 to modify the x axis |
| ChatGPT | Certainly! To modify the x-axis in ggplot2, you can use the scale_x_continuous function. Here's an example code snippet:  # Sample data (replace this with your own dataset)  set.seed(123)  data <- rnorm(100)  # Create a histogram with ggplot2 and modify the x-axis  hist_plot <- ggplot(data, aes(x = data)) +  geom_histogram(binwidth = 0.5, fill = "blue", color = "black", alpha = 0.7) +    # Add a vertical line for the median  geom_vline(xintercept = median(data), color = "red", linetype = "dashed", size = 1) +    # Modify the x-axis  scale_x_continuous(name = "Custom X-axis Label", # Change the x-axis label  breaks = seq(-3, 3, by = 1), # Adjust tick marks  limits = c(-3, 3)) # Adjust axis limits    # Add labels and a title  labs(title = "Histogram with Modified X-axis",  x = "Values",  y = "Frequency")  # Display the plot  print(hist_plot)  In this example, scale_x_continuous is used to customize the x-axis label, set custom breaks for tick marks, and adjust the axis limits. Modify the arguments within scale_x_continuous according to your specific needs. |
| Authors | Please write in english Dies führte dazu dass 0.55% aller HR Messungen in der Datenauswertung als nicht valide exkludiert wurden |
| ChatGPT | Translated to English:  "This resulted in the exclusion of 0.55% of all heart rate measurements in the data analysis as non-valid." |
| Authors | Please translate to english and correct if neccesary: Die Berechnung der Data completeness der HR Messungen selbst wurde berechnet in welchem Anteil der 20 Studientage eine Messung pro 15min aufgezeichnet wurde, eigentliches von den Herstellern angegebenes Messfenster: 10min. Dabei ergaben sich jedoch für einen Großteil der Teilnehmer keine bis kaum Messungen, 75%-Quantil: 5% Data completenss. Dies zeigt sich auch in der folgenden Abbildung mit Einzeichnung des Medians: |
| ChatGPT | Translated and corrected in English:  "The calculation of Data Completeness for the heart rate measurements was conducted to determine the proportion of the 20 study days during which a measurement was recorded every 15 minutes, contrasting with the manufacturer's specified measurement window of 10 minutes. However, for a significant portion of the participants, there were few to no measurements, with a 75th percentile Data Completeness of 5%. This is evident in the following figure, with the median marked:" |
| Authors | This is evident in the following figure, with the median marked:" please reformulate |
| ChatGPT | This is apparent in the accompanying figure, where the median is indicated. |
| Authors | Please translate and correct: Da in der Analyse der Validiät einzelne Momentaufnahmen der Herzfrequenzen untrschiedlciher personen mit interindividuellen hR unterschieden, die durchschnittswerte satrk beinflussen haben wir nur Personen mit 50% DC in der Analyse inkludiert |
| ChatGPT | Translated and corrected:  "In the analysis of validity, as single snapshots of heart rates varied among different individuals with interindividual heart rate differences that strongly influenced the average values, we included only individuals with 50% Data Completeness in the analysis." |
| Authors | Um in der Validitäts analyse ein möglichst abdeckendes Bild über die Herzfreqzenzmesungen gegeben werden soll, wurden nur die Daten von Teilnehmern mit mindestens 50% HR completeness inkludiert. translate and corrrect please |
| ChatGPT | Translated and corrected:  "To provide a comprehensive picture of heart rate measurements in the validity analysis, only the data from participants with at least 50% HR completeness were included." |
| Authors | füge noch an dass wir das machen da wir die durchschnittswerte, insbesondere den Tagesverlauf betrachten |
| ChatGPT | "To provide a comprehensive picture of heart rate measurements in the validity analysis, only the data from participants with at least 50% HR completeness were included. This approach was taken as we are particularly interested in examining average values, especially the daily patterns." |
| Authors | sind im appendix angefügt auf englisch bitte |
| ChatGPT | "Attached in the appendix." |
| Authors | Pleae to englisch: Eine ausführliche Übersicht über aller multiplen regressionsmodelle ist in Tabelle 1 dargestellt. Dort sind jeweils für die einzelenen Health parameter und die dazu in Zusammenhanggesetzten Extremwetter indikatoren, Temperatur, WBGT, Heat index und precicpitation Estimates, Standard errors und p-values gelistet. Außerdem sind auch die weiteren im Modell betrachteten confounder dargestellt. |
| ChatGPT | An extensive overview of all multiple regression models is presented in Table 1. For each health parameter and the associated extreme weather indicators, namely temperature, WBGT, heat index, and precipitation estimates, the table lists standard errors and p-values. Additionally, it includes the other confounders considered in the model. |
| Authors | je nach Model englsich |
| ChatGPT | Depending on the model. |
| Authors | Please correct and write in proper english if necessary: Regression analysis results for health parameters, using a logarithmically transformed model for step count and standard models for sleep duration and body shell. For each health parameter, namely sleep duration, step count and body shell temperature, and the associated extreme weather indicators, the table lists estimates, standard errors, and p-values. Weather indicators considered depending on the model were temperature, heat index, WBGT and precipitation. Additionally, the multiple linear regression models incorporated gender, age, and BMI as data validity analysis demonstrated these confounders to be of significance |
| ChatGPT | Corrected version:  "Regression analysis results for health parameters include a logarithmically transformed model for step count and standard models for sleep duration and body temperature. The table provides estimates, standard errors, and p-values for each health parameter—sleep duration, step count, and body temperature—along with their associated extreme weather indicators. Depending on the model, weather indicators considered were temperature, heat index, WBGT, and precipitation. Additionally, the multiple linear regression models incorporated gender, age, and BMI, as the data validity analysis demonstrated the significance of these confounders." |
| Authors | In english and please correct: In anderen Studien mit Withingsgeräten, hier jedoch mit älteren Modellen, haben ausserdem Fehlfunktionen der Wearables aufgrund von z.b. Verbindungsproblemen zu missing data geführt |
| ChatGPT | Corrected:  "In other studies using Withings devices, albeit with older models, malfunctions of the wearables, such as connection issues, have also led to missing data." |
| Authors | wie füge ich zu einem datum in r eine neue Spalte mit dem jeweiligen Wochentag ein? |
| ChatGPT | In R kannst du eine neue Spalte mit dem jeweiligen Wochentag zu einem Datum hinzufügen, indem du die Funktionen as.Date und weekdays verwendest. Hier ist ein Beispiel:  # Beispiel-Daten erstellen  dates <- c("2023-01-01", "2023-01-02", "2023-01-03", "2023-01-04")  # Spalte mit dem Datum erstellen  df <- data.frame(Date = as.Date(dates))  # Neue Spalte mit dem Wochentag hinzufügen  df$Weekday <- weekdays(df$Date)  # Ausgabe anzeigen  print(df)  In diesem Beispiel wird eine Spalte mit dem Namen "Weekday" erstellt, die die Wochentage für die entsprechenden Daten enthält. Du kannst dies an deine spezifischen Daten anpassen. |
| Authors | wie füge ich die weekdays in englisch hinzu |
| ChatGPT | Um die Wochentage in Englisch hinzuzufügen, kannst du die Funktion format verwenden, indem du das Argument "%A" verwendest. Hier ist ein Beispiel:  # Beispiel-Daten erstellen  dates <- c("2023-01-01", "2023-01-02", "2023-01-03", "2023-01-04")  # Spalte mit dem Datum erstellen  df <- data.frame(Date = as.Date(dates))  # Neue Spalte mit dem Wochentag in Englisch hinzufügen  df$Weekday <- format(df$Date, "%A")  # Ausgabe anzeigen  print(df)  In diesem Beispiel wird die Spalte "Weekday" mit den englischen Wochentagen erstellt. Du kannst dies für deine eigenen Daten anpassen. |
| Authors | die tage werden mir trotzdem auf deutsch angezeigt, kennst du noch eine andere Methode |
| ChatGPT | Entschuldigung für die Verwirrung. Wenn die Wochentage weiterhin auf Deutsch angezeigt werden, könnte dies an den Einstellungen deiner R-Umgebung liegen. Du kannst die Sprache für die Datumsformatierung explizit festlegen. Hier ist ein Beispiel:  # Beispiel-Daten erstellen  dates <- c("2023-01-01", "2023-01-02", "2023-01-03", "2023-01-04")  # Spalte mit dem Datum erstellen  df <- data.frame(Date = as.Date(dates))  # Sprache auf Englisch setzen und dann neue Spalte mit dem Wochentag hinzufügen  Sys.setlocale("LC_TIME", "English")  df$Weekday <- format(df$Date, "%A")  # Ausgabe anzeigen  print(df)  Die Funktion Sys.setlocale("LC_TIME", "English") setzt die Sprache für die Datumsformatierung auf Englisch. Danach wird die Spalte "Weekday" erstellt. Stelle sicher, dass deine R-Umgebung die englische Sprache unterstützt. |
| Authors | kannst du mir auch noch sagen wie ich nur anfüge ob es ein wochentag oder ein wochende ist? |
| ChatGPT | Natürlich! Du kannst eine neue Spalte erstellen, die angibt, ob es sich um einen Wochentag oder ein Wochenende handelt. Hier ist ein Beispiel:  # Beispiel-Daten erstellen  dates <- c("2023-01-01", "2023-01-02", "2023-01-03", "2023-01-04", "2023-01-07", "2023-01-08")  # Spalte mit dem Datum erstellen  df <- data.frame(Date = as.Date(dates))  # Sprache auf Englisch setzen  Sys.setlocale("LC_TIME", "English")  # Neue Spalte mit dem Wochentag hinzufügen  df$Weekday <- format(df$Date, "%A")  # Neue Spalte für Wochentag oder Wochenende erstellen  df$DayType <- ifelse(weekdays(df$Date) %in% c("Saturday", "Sunday"), "Weekend", "Weekday")  # Ausgabe anzeigen  print(df)  In diesem Beispiel wird eine neue Spalte "DayType" erstellt, die angibt, ob es sich um einen Wochentag oder ein Wochenende handelt. ifelse wird verwendet, um zu überprüfen, ob der Wochentag Samstag oder Sonntag ist. Du kannst dies an deine spezifischen Anforderungen anpassen. |
| Authors | kannst du mir einen knackigen englsichen überbegriff für wochenende und wochentage geben? |
| ChatGPT | Natürlich! Hier sind knackige englische Begriffe für "Wochenende" und "Wochentage":   - Wochenende: "Weekend" - Wochentage: "Weekdays" |
| Authors | Nee so als überbegriff wie Wochenabschnitt |
| ChatGPT | Ah, ich verstehe. Ein knackiger Überbegriff könnte sein:   - "Weektime" (für Wochentage) - "Weekend Block" (für Wochenende und Wochenanfang)   Es gibt keine genauen direkten Übersetzungen, aber diese Begriffe könnten als Überbegriffe für Wochentage und Wochenenden dienen. Du kannst sie an deine spezifischen Anforderungen anpassen. |
| Authors | Please translate to english: Intraindividuelle Unterschiede wurden in dieser Studie aufgrund der nur kurzen Wearables-Tragedauer der einzelnen Teilnehmer von 21 Tagen nicht betrachtet, da intraindividuelle Schwankungen statistisch nicht sicher als Abweichung oder Norm einordbar sind. Zur Betrachtung der Effekte über die unterschiedlichen Wochentage hinweg haben wir in einer zusätzlichen Analyse den möglichen zeitlichen Confounder Wochentag bzw. Wochenende hinzugefügt, dieser zeigte jedoch in keinem der Modelle einen signifkatnten Einfluss (p>0.05). Die genauen Ergebnisse dieser Analyse sind im Appendix XXX zu finden. |
| ChatGPT | Individual differences were not considered in this study due to the short duration of wearables usage by each participant, limited to 21 days. This decision was made because statistically, intra-individual variations cannot be confidently classified as deviations or norms within such a brief timeframe. To assess effects across different weekdays, we included a potential temporal confounder, namely, weekday or weekend, in an additional analysis. However, this confounder did not exhibit a significant impact in any of the models (p > 0.05). Detailed results of this analysis can be found in Appendix XXX. |
| Authors | und zusätzlich dazu englisch |
| ChatGPT | Intraindividual differences were not considered in this study due to the relatively short wearables usage duration of each participant, limited to 21 days. Statistical uncertainties in categorizing intra-individual fluctuations as deviations or norms within such a brief timeframe led to this exclusion. To examine effects across different weekdays, we included a potential temporal confounder, namely, weekday or weekend, in an additional analysis. However, this confounder did not show a significant impact in any of the models (p > 0.05). The detailed results of this analysis can be found in Appendix XXX. |
| Authors | kannst du mir verscheidene englische optionen nennen um "und zusätzlich dazu" asuzudrücken |
| ChatGPT | Sicher, hier sind verschiedene Möglichkeiten, "und zusätzlich dazu" auf Englisch auszudrücken:   1. "Moreover," 2. "Furthermore," 3. "In addition," 4. "Additionally," 5. "Also," 6. "Besides," 7. "Furthermore, it should be noted that," 8. "Moreover, it is important to mention that,"   Je nach Kontext kannst du die am besten passende Option wählen. |
| Authors | was heisst likely auf deutsch |
| ChatGPT | "Likely" bedeutet auf Deutsch "wahrscheinlich" oder "vermutlich". |
| Authors | please translate and reformulate properly to english: ⎝ Eine Studie die sich die self-reported Schlaf während den Covid Lockdowns in 2020 angeschaut hat, hat bei der Mehrheit der Teilnehmenden eine verkürzte gesamtschlafdauer festgestellt während des lockdowns |
| ChatGPT | "A study examining self-reported sleep during the Covid lockdowns in 2020 found that the majority of participants experienced a shortened total sleep duration during the lockdown." |
| Authors | and add: bei jedoch einer zunahme an naps |
| ChatGPT | "A study examining self-reported sleep during the Covid lockdowns in 2020 found that the majority of participants experienced a shortened total sleep duration during the lockdown, accompanied by an increase in naps." |
| Authors | Please to english: Und eine weitere study die sich exemplarisch Mobilität während Covid19 in Südafrika angeschaut hat, hat einen Rückgang dieser, vorallem an Wochenden beobachtet. |
| ChatGPT | "And another study that focused on mobility during Covid-19 in South Africa observed a decline, especially on weekends." |
| Authors | Mehrere Schlafepisoden von >3h wurden zusammengerechnet wenn der Beginn der letzen Episode vor 12 Mittags des nächsten tages war |
| ChatGPT | "Multiple sleep episodes of >3 hours were aggregated if the start of the last episode was before 12 noon of the next day." |
| Authors | das bitte auf english |
| ChatGPT | "Multiple sleep episodes of >3 hours were combined if the start of the last episode occurred before 12 noon of the next day." |
| Authors | Bitte einmal alles in englisch und ordentlich formulieren: Vom Wearable gemessene Wake time wurde nicht in die sleep duration inkludiert und Split sleep of >3 hours were aggregated if the start of the last episode was before 12 noon of the next day |
| ChatGPT | "The wake time measured by the wearable was not included in the sleep duration. Split sleep episodes of >3 hours were combined if the start of the last episode occurred before 12 noon of the next day." |
| Authors | Please to englishc and correct if necessary: Jedoch muss beachtet werden, dass sowohl Kleidung, Luftzirkulation, Bettwäsche aber auch individuelle Einflüsse wie das Geschlecht einfluss auf Assoiation von Hitze und Schlafdauer haben, und in dieser Studie nicht berücksichgtigt wurden |
| ChatGPT | "However, it must be noted that factors such as clothing, air circulation, bedding, and individual influences like gender can affect the association between heat and sleep duration, and were not considered in this study." |
